# Transat—A Method for Detecting the Conserved Helices of Functional RNA Structures, Including Transient, Pseudo-Knotted and Alternative Structures

**DOI:** 10.1371/journal.pcbi.1000823

**Published:** 2010-06-24

**Authors:** Nicholas J. P. Wiebe, Irmtraud M. Meyer

**Affiliations:** Centre for High-Throughput Biology & Department of Computer Science and Department of Medical Genetics, University of British Columbia, Vancouver, British Columbia, Canada; Bar-Ilan University, Israel

## Abstract

The prediction of functional RNA structures has attracted increased interest, as it allows us to study the potential functional roles of many genes. RNA structure prediction methods, however, assume that there is a unique functional RNA structure and also do not predict functional features required for *in vivo* folding. In order to understand how functional RNA structures form *in vivo*, we require sophisticated experiments or reliable prediction methods. So far, there exist only a few, experimentally validated transient RNA structures. On the computational side, there exist several computer programs which aim to predict the co-transcriptional folding pathway *in vivo*, but these make a range of simplifying assumptions and do not capture all features known to influence RNA folding *in vivo*. We want to investigate if evolutionarily related RNA genes fold in a similar way *in vivo*. To this end, we have developed a new computational method, Transat, which detects conserved helices of high statistical significance. We introduce the method, present a comprehensive performance evaluation and show that Transat is able to predict the structural features of known reference structures including pseudo-knotted ones as well as those of known alternative structural configurations. Transat can also identify unstructured sub-sequences bound by other molecules and provides evidence for new helices which may define folding pathways, supporting the notion that homologous RNA sequence not only assume a similar reference RNA structure, but also fold similarly. Finally, we show that the structural features predicted by Transat differ from those assuming thermodynamic equilibrium. Unlike the existing methods for predicting folding pathways, our method works in a comparative way. This has the disadvantage of not being able to predict features as function of time, but has the considerable advantage of highlighting conserved features and of not requiring a detailed knowledge of the cellular environment.

## Author Summary

Many non-coding genes exert their function via an RNA structure which starts emerging while the RNA sequence is being transcribed from the genome. The resulting folding pathway is known to depend on a variety of features such as the transcription speed, the concentration of various ions and the binding of proteins and other molecules. Not all of these influences can be adequately captured by the existing computational methods which try to replicate what happens *in vivo*. So far, it has been challenging to experimentally investigate co-transcriptional folding pathways *in vivo* and only little data from *in vitro* experiments exists. In order to investigate if functionally similar RNA sequences from different organisms fold in a similar way, we have developed a new computational method, called Transat, which does not require the detailed computational modeling of the cellular environment. We show in a comprehensive analysis that our method is capable of detecting known structural features and provide evidence that structural features of the *in vivo* folding pathways have been conserved for several biologically interesting classes of RNA sequences.

## Introduction

RNA molecules play diverse roles in many of the most basic cellular processes. In the translation process, for instance, the protein coding ‘message’ is encoded in a messenger RNA (mRNA) and transfer RNAs (tRNAs) and ribosomal RNAs (rRNAs) are involved in this catalytic process. Micro RNAs are implicated in regulating mRNA availability. A range of other non-protein-coding RNAs (ncRNAs) have been identified [1], [2]. Moreover, studies of mammalian transcriptomes have found, rather surprisingly, that the majority of the genome is transcribed, and that the vast majority of transcripts do not overlap with known protein-coding regions, hinting at the possibility that many functionally important classes of ncRNAs remain to be discovered [2], [3].

For many classes of ncRNA molecules studied so far, RNA structure plays a crucial part in defining its functional role in the cell. We know, for example, that tRNAs assume a distinct three-dimensional conformation in order to function properly during translation and that the functional configuration of the ribosome complex relies both, on properly folded rRNAs as well as many proteins binding to the respective rRNAs. In contrast to proteins, we can typically learn a lot about an RNA's functionality by studying only its secondary structure, i.e. the set of base-pairing nucleotide positions in the RNA sequence. This is the case because most RNA sequences studied so far fold in a hierarchical manner, with the secondary structure emerging first and the tertiary contacts between secondary structure elements emerging later.

*In vivo*, an RNA molecule is synthesized during transcription and will immediately start to fold [4], [5]. A succession of cellular events — involving, for example, splicing, RNA editing, the binding of proteins, metabolites or other RNA molecules — may influence the kinetic, co-transcriptional folding pathway *in vivo* which yields one or more biologically active, i.e. functional structural confirmations.

The view that one RNA sequence has one functional RNA structure turns out to be too simplistic. We know by now of several cases, where a given RNA sequence has more than one functionally important RNA structure, e.g. ribo-switches [6]–[8] which change their structure upon binding a metabolite, as well as cases, where a transient RNA structure is functionally important [9], [10]. We therefore propose to develop a method which allows us to identify evolutionarily conserved structural elements which are likely to be required for the formation of the functional structures *in vivo*.

There exist by now a wide range of computational methods that can predict an RNA secondary structure given an RNA sequence. Many of these methods [11]–[14] in particular earlier methods, aim to predict the thermodynamically most stable RNA secondary structure. Many biological systems, however, are not in thermodynamic equilibrium. The predictions of these so-called minimum-free energy (MFE) methods depend on the underlying energy parameters which in turn depend on the temperature, the ion concentration and other parameters. Theoretical studies of RNA molecules [15] have shown that the thermodynamic structure of even moderately long RNA molecules often does not correspond to the functional RNA structure that has been conserved during evolution, i.e. the RNA structure that exerts the biological function *in vivo*. This may, at least partly, be due to co-transcriptional folding [4], [5], [16]. More recent structure prediction methods use a comparative approach which simultaneously analyzes several evolutionarily related RNA sequences from different organisms [17]–[32]. Detailed structural studies employing several dedicated evolutionary models [21] find that the substitution rate in base-paired regions is reduced by a factor of 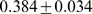 and in loop and bulges by a factor of 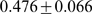 with respect to the substitution rate in un-structured regions, i.e. that loops and bulges tend to evolve significantly slower than un-structured regions and only slightly faster than base-paired regions at least in set of RNA structures investigated in [21]. This is in line with our expectation that loops and bulges are on average more likely to be bound by other molecules (RNAs, DNAs or proteins) than unstructured regions. These comparative methods aim to detect the RNA secondary structure that has been conserved during evolution. The implicit assumption made by these methods is that evolutionarily conserved structures are likely to be functionally important which has been shown to be a reasonable assumption. The performance of these comparative methods is – generally speaking – higher than that of non-comparative methods [33] provided the input data are high-quality multiple-sequence alignments or the method is capable of generating a multiple-sequence alignment as part of its predictions [27]–[30], [32]. All of the above computational methods, however, only aim to predict a single RNA structure and cannot be used to detect the presence of transient RNA structures or the presence of multiple functional RNA structures such as, for example, ribo-switches which are known to have two distinct functional structures.

The program RNAsubopt [34] takes a single RNA sequence as input and predicts a list of all structures below a certain energy cutoff. Enumerating enough structures to capture most of the structure probability, however, is only possible for short sequences. Moreover, since the total number of possible structures is so vast, the probability of any particular structure is not a reliable indicator for identifying potential alternative structures. Rather, one would like to group similar structures together, and identify groups with a high overall probability. Voss *et al.* [35] formalize this grouping process by defining abstraction functions in order to map structures to ‘RNA shapes’, and are capable of calculating the total probability for a given shape. The runtime for this method grows exponentially with sequence length, making it impractical for sequences longer than about 400 nucleotides [36]. It is possible, however, to sample structures from the Boltzmann distribution in polynomial time [37], and to then apply the RNA shapes abstraction in order to estimate the shape probability for longer sequences. This approach is capable of recovering alternative structures for some ribo-switches [35]. All of the above approaches assume the RNA sequence to be in thermodynamic equilibrium and are thus limited to identifying alternative structures which occupy a significant portion of the Boltzmann distribution. For co-transcriptionally folding RNA sequences (which may become kinetically trapped), this assumption does not necessarily hold and the time-averaged probabilities for different structural configurations encountered during the kinetic folding may differ markedly from their respective probabilities derived from the Boltzmann distribution.

*In vivo*, RNA molecules are known to fold co-transcriptionally [4], [5], i.e. while they emerge during transcription. The resulting kinetic folding pathway can depend on a variety of events during and after transcription such as the speed of transcription [9], [38], [39], splicing [40], RNA editing [41], the binding of proteins [42], metabolites [43] and other RNA molecules [44], the temperature and the concentrations of monovalent and divalent ions [45]. The co-transcriptional folding pathway can differ significantly from the re-folding one [46], [47], both in terms of time line and structural features.

The increasing interest in RNA folding pathways has spurred the development of computational methods for RNA structure prediction which take the folding kinetics explicitly into account. These methods try to model the physical process by which an unfolded RNA folds into its functional conformation(s) as a continuous-time Markov process which allows only local rearrangements of secondary structures. If we knew all entries of the transition rate matrix 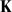 containing the transition rates between all pairs of possible structures, the vector of probabilities for all structures at a given time could be calculated as 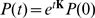. As the state space of all possible secondary structures can be very large for RNAs of biological interest, it is generally not feasible to calculate the full transition matrix. However, folding trajectories can be sampled using Monte Carlo stochastic simulation of the Markov process. Several programs, including RNAkinetics [48]–[50], Kinfold [51] and Kinefold [52]–[54], employ this method, though they differ significantly in their implementation.

Mironov and Lebedev [49] were the first to model the co-transcriptional folding of an emerging RNA sequence and to allow entire helices not only to form, but also to disintegrate [55]. The transition probabilities of their Markov chain Monte-Carlo method correspond to the chemical rate constants for forming and disintegrating helices [48] and thus have a clear physical interpretation. Their theoretical framework could be readily extended to also deal with pseudo-knotted RNA secondary structures [49].

Kinfold [51] defines legal transitions as the formation, disruption, or shifting of a single base-pair. The folding trajectories it generates are therefore very fine-grained, specifying when each base-pair is added or removed. In Kinefold [52]–[54], transitions add or remove entire helices, a simplification which reduces the number of legal transitions from any state, but which also requires a more complex estimation of the transition state energy. The program assumes that the energy barrier is the energy required to nucleate three base pairs of a new helix, plus the energy required to displace any helices blocking the formation of the new helix. Kinefold also allows for pseudo-knotted structures, which requires a more complicated energy model than the standard Turner model [56] used by Kinfold (which ignores pseudo-knots). Kinefold also takes into account some topological constraints induced by pseudo-knots which may kinetically trap other helices [54]. Both programs can simulate the folding from an unfolded state as well as the co-transcriptional folding of an emerging sequence. The latter is done by dividing the sequence into transcribed and un-transcribed regions whose boundary shifts 5′ to 3′ at a certain rate, and restricting legal moves to those that form no base-pairs in the un-transcribed region. Neither of these two programs can model dynamic transcription speeds, although there is experimental evidence that transcriptional pausing influences the folding [57].

Other computational approaches for predicting kinetic folding pathways consider energy landscapes in order to reduce the size of the state-space. The energy landscape can be viewed as a barrier tree, where the local minima are leaves in the tree which are connected to one or more gradient basins via saddle-points. Saddle-points are the lowest energy structures that connect the gradient basins around these local minima [51], [58]. Constructing such a barrier tree representation of the energy landscape requires the consideration of all possible structures. Barrier trees constructed from a list of the lowest-energy structures (generated with RNAsubopt [34]) typically capture the most relevant features of the energy landscape for sufficiently short sequences (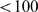 base pairs (bp)). In order to reduce the state space, Wolfinger *et al.* [59] define the state-space as the basins around local minima of the energy landscape, and calculate the transition rates between adjacent basins using a variation of the so-called flooding algorithm used to construct barrier trees. Barrier trees are also useful for interpreting folding trajectories sampled with Monte Carlo simulations [51]. A similar approach is taken by Tang *et al.* [60], [61], where the folding landscape is approximated by a probabilistic road map which defines the allowed transitions between states. They restrict the state-space to a set of secondary structures probabilistically sampled from the Boltzmann distribution. Transitions are only allowed to the nearest 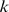 neighbors, with energy barriers estimated heuristically. Ideally, these states should capture the main features of the folding landscape while being few enough to solve the master equation (though it is also possible to do Monte Carlo simulation here). Zhang and Chen [62]–[64] partition the structure space into clusters based on the presence or absence of certain (somewhat arbitrarily chosen) rate-limiting base-stacks, which have particularly high energy barriers to their formation or disruption. The distribution of structures within clusters is assumed to be at thermodynamic equilibrium, so the transition rates between clusters can be calculated by summing the rates of transition between the structures at the boundaries of clusters, adjusted for the probability of the boundary structure in its cluster. All of these thermodynamic-landscape-based methods, however, are not applicable to the analysis of co-transcriptional folding, since an RNA's energy landscape changes while it is being transcribed. By calculating energy landscapes for all partially transcribed subsequences and then mapping the local minima from each landscape onto its successor, however, one could – in theory – adapt landscape-based methods to co-transcriptional folding [65].

Long sequences are problematic for all the above methods since the number of possible secondary structures, and therefore the worst-case complexity of the energy landscape, grows exponentially with the sequence length. The Kinwalker program [66] was designed to allow the analysis of the folding kinetics for long sequences (around 1000 bp). For this, it dispenses with simulation and instead deterministically predicts a potential co-transcription folding pathway which is pieced together from heuristically chosen combinations of pre-computed minimum free-energy (MFE) structures for short sub-sequences and assumes (similar to MFE methods for RNA structure prediction) the pseudo-knot free MFE structure to be the final RNA structure. The method can be considered kinetic in that it allows the incorporation of an MFE sub-structure only if the energy barrier between the current structure and the resulting merged structure that the transition can occur within a reasonable time, i.e. before the next transcription step. Calculating the energy barrier between two arbitrary structures, however, has been shown to be NP-complete [67]. Kinwalker thus employs a further heuristic for estimating these barriers. In summary, Kinwalker aims to find the MFE structure at each transcription step, subject to the constraint that the transitions between structures be kinetically feasible.

All of the above prediction methods take at most the RNA sequence itself, the temperature, the 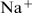 concentration and a constant transcription speed into account, but do not capture any potential interactions with other molecules or other features of the biological environment which may influence the folding pathway *in vivo*. The latter is difficult to do, not only because we typically lack information on the interaction partners and the mechanisms and timing of their interactions, but also because we cannot easily capture the wealth of relevant details of the complex cellular environment in a computationally tractable model. The performance of the existing computational methods can strongly depend on the sequence length and other features of the individual input sequence. This is not surprising given that any errors in the early stages of the folding pathway prediction are magnified as the folding progresses. A precise knowledge of the transcription start site i.e. the 5′ end of the RNA sequence is thus crucial. The prediction performance of the existing methods has thus only been evaluated on very small data sets.

It is also challenging to study kinetic folding pathways experimentally. There exist by now a range of powerful experimental techniques for studying large sets of RNA sequences in an ensemble-averaged way such as UV melting, isothermal titration calometry, circular dichroism, chemical foot-printing and, more recently, single-molecule techniques such as fluorescence correlation spectroscopy [68], single-molecule fluorescence resonance energy transfer [69] and force spectroscopy [70], [71]. These experimental methods, however, still await to be taken from the test tube to the cell in order to explore how RNA sequences fold *in vivo* [72].

We propose a conceptually new computational approach for studying RNA folding pathways *in vivo*. Rather than trying to replicate the folding kinetics of a single RNA sequence *in vivo* — which is very difficult to do — we introduce a comparative approach which takes several evolutionarily related RNA sequences as well as an evolutionary tree relating these sequences as input. Our main goals in devising Transat can be summarized as follows:

- predict evolutionarily conserved helices that are likely to play a role in the co-transcriptional formation of the functional RNA structure(s) *in vivo*
- do not require a detailed knowledge of the *in vivo* environment, e.g. transcriptional speed, ion concentrations, interaction partners etc., and keep the number of free parameters and assumptions incorporated into the method to a minimum
- estimate reliability values for all predictions
- present a comprehensive performance evaluation
- have a performance which is robust with respect to sequence length

## Methods

### The prediction program Transat

#### Motivation

If a structural feature is functionally important, it is typically well conserved in groups of related RNAs, even if the level of primary sequence conservation may be low. These conserved structural features can be detected in alignments of several evolutionarily related RNA sequence by identifying pairs of alignment columns where the base-pairing potential, but not necessarily the primary sequence itself has been conserved. This analysis of these so-called co-varying alignment columns is even capable of identifying tertiary structure motifs [73], [74]. Functionally equivalent RNA sequences from related organisms tend to be more conserved in terms of RNA structure than primary sequence, making it often challenging to establish high-quality sequence alignments based on primary sequence conservation only. Theoretically, all functional helices, regardless of their stability or transience, should be evolutionarily conserved (though not necessarily equally conserved), and comparative methods should therefore be capable of identifying functional transient or alternative structures. Moreover, evolution acts on *in vivo* structures, which may be influenced by protein-binding, RNA binding in *trans* or other local factors. These interactions would need to be taken into account by non-comparative methods which try to replicate the co-transcriptional folding process, but are currently ignored. Comparative methods, however, do not necessarily require information on interaction partners because they derive their predictions from the observed patterns of covariation. One can even argue that comparative methods may be able to *predict* single-stranded binding sites by identifying regions which are devoid of conserved structural features.

We have devised Transat as a comparative method which takes as input a fixed multiple-sequence alignment of evolutionarily related RNA sequences from different organisms and an evolutionary tree relating these sequences. It employs a multiple step strategy in order to predict a set of conserved helices that can be considered statistically significant.

#### Identifying conserved helices and calculating their log-likelihood values

**Figure 1:**
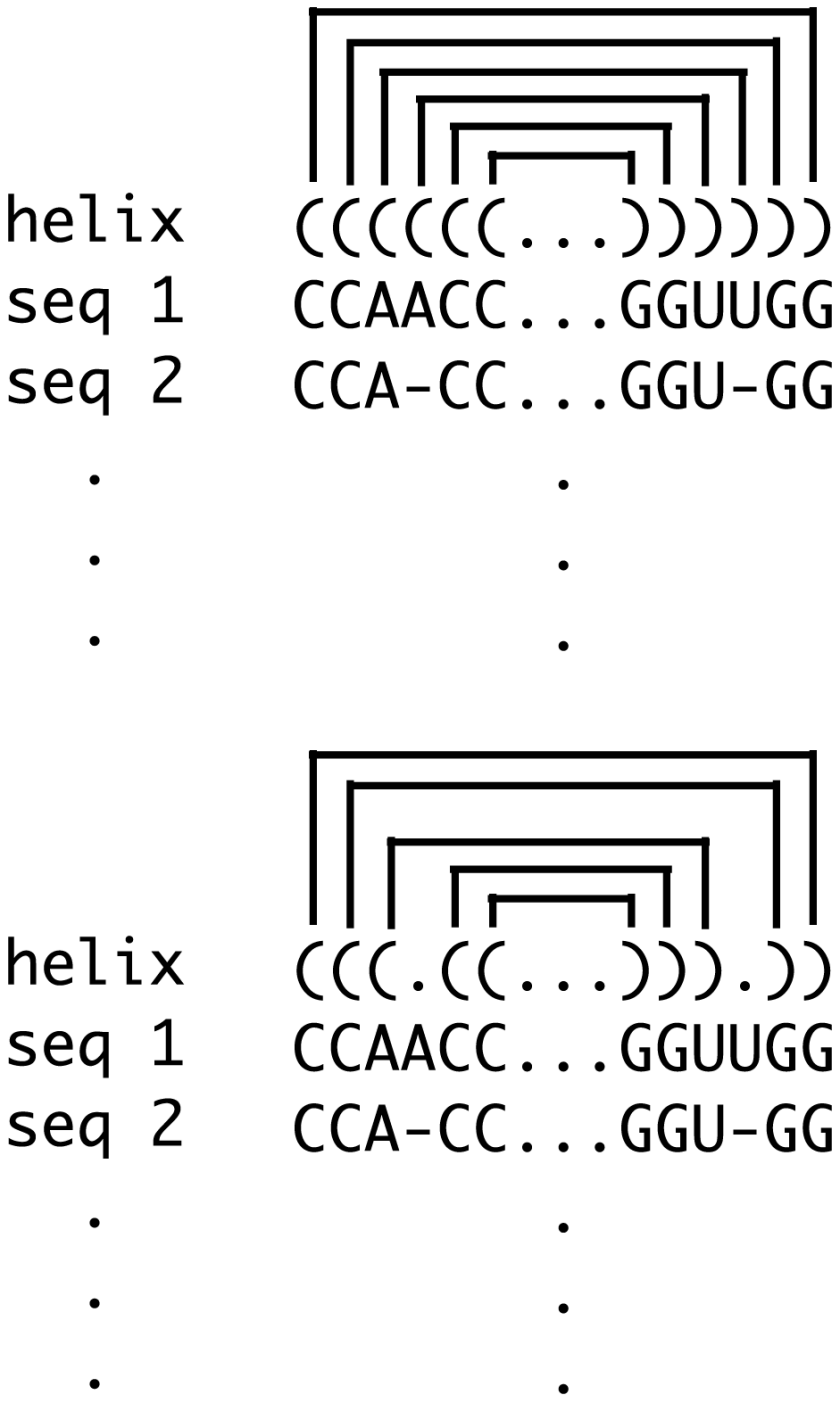
Transat: Mapping of helices to the alignment. Transat first predicts the helices for all individual sequences in the fixed input alignment and then maps all of them to the alignment remembering the base-pairing sequence positions. In the example above, there are two helices, one derives from sequence 1 (see top figure), the other one from sequence 2. Mapping these two helices from their respective sequence to the entire alignment results in the two potential conserved helices shown above (see the arcs linking the respective alignment columns). Both conserved helices are then evaluated by Transat in terms of log-likelihood value and p-value estimation. The log-likelihood value is calculated based on the base-paired alignment columns in that helix and all sequences in the alignment, see the text and Figure 2 for details. All helices predicted by Transat for a given input alignment can then be ranked according to their p-value. For the two helices in the example above, the helix that fits the sequences in the given alignment better will have the higher log-likelihood value and lower p-value. As Transat is not capable of modifying the fixed input alignment, this mapping strategy minimized the impact of alignment errors.

**Figure 2:**
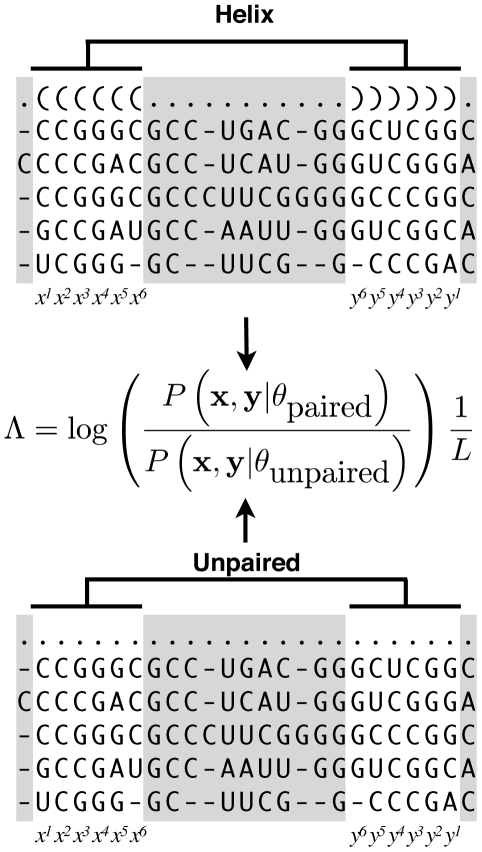
Log-likelihood calculation for a conserved helix detected by Transat. See the text for more details.

In the first step, Transat determines helices for each individual, un-gapped sequence of the input alignment. We define a helix to consists of 4 or more consecutive consensus base-pairs which are 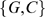, 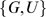 and 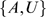. In the next step, the helices of the individual sequences are mapped onto the input alignment, see Figure 1 for details. This procedure produces many helices spanning *all* sequences of the multiple sequence alignment and ensures that the impact of alignment errors is minimized. We call these helices conserved helices. For each conserved helix 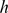, we then compute the log-likelihood score as follows:
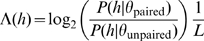
where 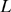 is the length of the helix in base-pairs, 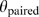 corresponds to the hypothesis that the alignment columns of 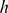 are base-paired and 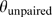 to the hypothesis that the alignment columns of 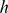 are un-paired, see Figure 2. Division by 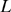 ensures that the log-likelihood scores are length-normalized.

We model the evolution of base-paired and un-paired alignment columns along the evolutionary input tree with two reversible, time-continuous Markov chains using the same rate matrices and equilibrium distributions as the comparative RNA structure prediction programs Pfold [20] and SimulFold [32]. The likelihood values 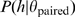 and 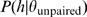 are both calculated using the Felsenstein algorithm [75] by taking only the alignment columns of the conserved helix 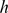 into account. If we hypothesize that the alignment columns of 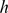 are base-paired, the overall likelihood is equal to the product of the likelihood values for all pairs of base-paired alignment columns, i.e. 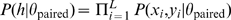. If we, however, hypothesize that the alignment columns of 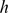 are unpaired, the overall likelihood is equal to the product of the likelihood values for all unpaired alignment columns, i.e. 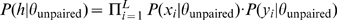. For two base-paired alignment columns 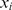 and 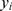, the corresponding likelihood is calculated using the Felsenstein algorithm and 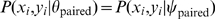, where 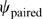 is an evolutionary model for base-paired alignment columns of length 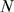, if 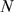 denotes the number of sequences in our multiple sequence alignment. Similarly, the likelihood for an unpaired alignment column 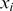 is also calculated using the Felsenstein algorithm and 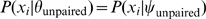, where 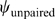 is an evolutionary model for unpaired alignment columns of length 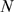. Each evolutionary model 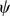 for alignment columns of length 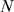 corresponds to a five-tuple 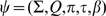, where 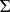 is the corresponding alphabet, 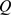 is the rate matrix, 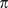 the vector of equilibrium frequencies, 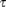 is a binary rooted tree topology and 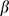 is a vector of branch lengths. 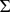, 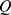 and 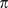 define a continuous Markov process which models the substitution process (either for paired or unpaired alignment columns) along the tree defined by 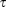 and 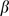. Either hypothesis, 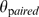 and 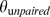, is thus captured by a probabilistic model of evolution. The Felsenstein algorithm [75] is a recursive algorithm which calculates the likelihood by moving from the leaf nodes of the evolutionary tree, i.e. the observed nucleotides and gaps in the corresponding alignment column or pair of alignment columns, via the internal tree nodes to the root node of the tree.

In contrast to the customary way of calculating the likelihood, we interpret one-sided gaps in base-paired alignment columns as non-consensus base-pairs rather than missing information. Two-sided gaps, however, are still treated as missing information which amounts to summing over all possible base-pairs when moving “up the tree” in the Felsenstein calculation. This treatment of two-sided gaps makes sense as the length of a helix can shrink or expand over time [76]. One-sided gaps, however, cannot be interpreted as the loss or gain of an entire base-pair and we therefore regard them as non-consensus base-pair.

In the likelihood calculation for two base-paired alignment columns, the Felsenstein algorithm traverses the tree from the leaf nodes (i.e. the observed nucleotides in two base-paired alignment columns) via the internal nodes to the root node of the tree. It sums over *all* possible nucleotide pairs at the internal nodes, weighing each possibility according to the corresponding entry of the pair rate matrix. If we interpret a gap in the base-pair 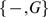 as missing information (as is customary), the Felsenstein algorithm takes *all* base-pairs, i.e. 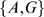, 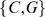, 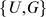 and 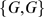, probabilistically in account at the corresponding *leaf node* thereby including two consensus pairs (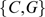 and 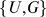). The likelihood of going from 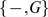 to the next internal tree node is dominated by the two good options, whereas we argue that it is conceptually more appropriate to interpret the gap as character which cannot base-pair with the other nucleotide (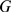 in this case). This is also in line with what we know about the evolution of RNA secondary structure, namely that helices tends to lose or acquire entire base-pairs, not half-pairs. Using our modified likelihood calculation which treats one-sided gaps as non-consensus base-pairs rather than missing information significantly increases our ability to distinguish base-paired from un-paired alignment columns.

#### Estimating p-values

The ability of an RNA sequences to form random helices is known to strongly depend on the sequence itself, in particular its length and its nucleotide and di-nucleotide composition. The log-likelihood value 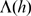 alone is thus typically not a reliable indicator of whether or not a helix 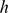 should be considered real. In order to correct for the fact that different RNA sequences have a higher chance of forming random helices than others, we estimate the p-value for the log-likelihood value of each conserved helix. This estimation procedure is done as follows for each input alignment separately.

In the first step, the input alignment is realigned based primary sequence conservation only using T-Coffee [77]. The purpose of the realignment step is to remove patterns that are only supported by secondary structure conservation and patterns that may have been introduced by human experts. The realigned alignment is then randomized following the procedure described by Washietl and Hofacker [78]. This involves first binning the alignment columns according to their primary sequence similarity and gap composition and then swapping alignment columns only within bins. This procedure ensures that a column can only be swapped for another column with a similar gap pattern and level of sequence conservation.

**Figure 3:**
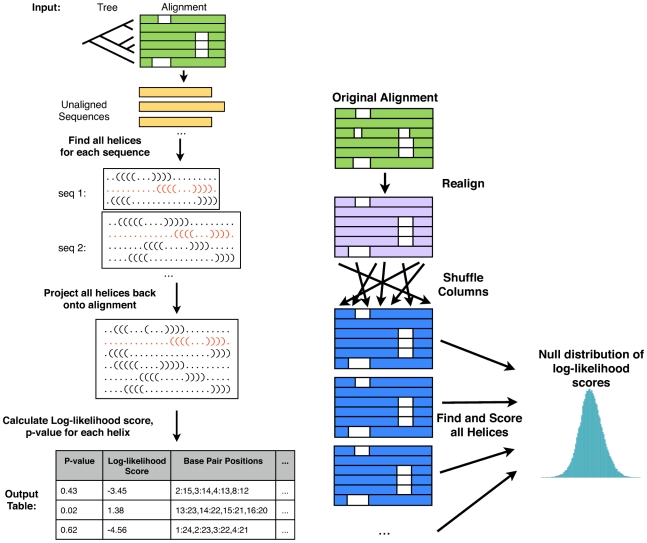
Overview of strategy employed by Transat. Transat takes as input a multiple sequence alignment and an evolutionary tree (left figure, top). It first predicts helices for all individual sequences in the alignment and then projects them back onto the multiple sequence alignment. It then calculates the log-likelihood value for each helix and estimates a p-value. The p-value estimation is explained in the right figure. In the first step, the original Transat input alignment is realigned based on primary sequence conservation only. In the second step, the columns of the resulting alignment are permuted multiple times, resulting in 500 shuffled versions of the original alignment. For each shuffled alignment, conserved helices are detected and their log-likelihood values calculated as for the original alignment. In the final step, the log-likelihood values of all helices in the shuffled alignments are entered into a histogram which is then used to derive p-values for the helices of the original alignment.

For each original alignment, we generate 500 randomized alignments. For each shuffled alignment (which we assume to no longer contain any real helices), we detect “conserved” helices that may have appeared by chance and then calculate their log-likelihood values. Both are done in the same way as for the original input alignment. We then combine the log-likelihood values from all 500 randomized alignments into a single histogram of log-likelihood values and use the resulting distribution to assign p-values to the log-likelihood values of the conserved helices in the original input alignment. Conserved helices in alignments with a high structure-formation potential thus require – generally speaking – larger log-likelihood values in order to be considered significant than helices in alignments where the overall structure-formation potential is lower. Figure 3 summarizes the strategy employed by Transat. The number of randomized alignments that it is to be generated for each input alignment to Transat is an input parameter to one of the programs of the Transat software package that can be easily adjusted by the user. The number of randomized alignments should be increased if significantly lower p-values are to be studied.

#### Output of Transat and brief summary

The output of Transat thus consists of a list of conserved helices and their corresponding log-likelihood and p-values. The user of Transat can then discard all conserved helices above a desired p-value threshold or rank the helices according to their p-values. For an input alignment of 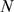 sequences and length 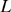, Transat requires 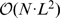 time. Transat does not require a known RNA secondary structure or known structural features for generating its predictions and makes only a few basic assumptions, namely how individual base-paired and unpaired nucleotides evolve (these assumptions are incorporated in the Felsenstein calculation) and what the number of consecutive base-pairs in a helix is (which we set to 4 and which can be changed).

## Results

### Datasets

Our data set comprises four sub-sets which have been chosen to represent (a) data, where multiple functional RNA secondary structures are known, (b) data, where only one functional RNA secondary structure is known and (c) artificially generated data which allows us to investigate some features of Transat in greater detail. Our aim was to compile a large and diverse data set and to include as many examples of known functional and transient RNA structures as possible. Taken together, our data set comprises 1126 multiple-sequence alignments whose lengths ranges from 100 to 1247 bp and which comprise between 6 and 712 sequences.

#### The Rfam data set

The Rfam database [79]–[81] contains multiple sequence alignments for a wide variety of RNA gene families. For each family, Rfam stores a manually curated seed alignment and a single conserved RNA secondary structure structure. These seed alignments are used in the Rfam database to generate a covariance model [82], [83] for each family. Each covariance model is a probabilistic model which captures structural as well as sequence features of the seed alignment and which can be used to search sequence (not structure) databases for RNA sequences that share both sequence *and* structural features with the corresponding RNA family. Each covariance model in Rfam is also used to compile a so-called full Rfam alignment which consists of the sequences in the seed alignments as well as additional nucleotide sequences from EMBL [84] that score above a certain threshold with the covariance model. As searching the entire EMBL data base with a covariance model would be too time-consuming, the data base is first pre-filtered by removing all sequences that lack a high-scoring Blast hit to at least one of the sequences in the seed alignment.

We select a sub-set of high-quality seed alignments from the Rfam database version 9.1 [81] which meet four criteria: (1) to consist of at least 5 sequences, (2) to have a minimum length of 100 bp, (3) to have a mean fraction of canonical base pairs larger than 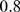 and (4) a covariation of at least 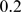 [85]. The mean fraction of canonical base-pairs corresponds to the proportion of consensus base-pairs in the base-paired alignment columns of the consensus structure. The closer this fraction is to 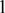, the better the consensus structure is supported by all sequences in the seed alignment. The covariation measures the fraction of base-paired alignment columns that are supported by mutations which maintain the base-pairing ability, but alter the nucleotides forming the base-pair.

Applying these four selection criteria, we arrive at a data set of 134 seed alignments which contain 6 to 712 sequences (average is 60 sequences) and whose length ranges from 100 to 1247 bp (average is 221 bp). The total tree length of these alignments ranges from 0.4 to 116.3, the average being 10.0. We call this data set the Rfam data set.

#### The artificial data set

In order to be able to investigate the dependence of the performance on the alignment length and the total tree length in detail, we generate an artificial data set comprising a total of 990 alignments. Each alignment in this artificial dataset is generated as follows. In the first step, an RNA secondary structure from the RNA STRAND database [86], a total tree length, and a desired number of sequences in the alignment is chosen. In the second step, a balanced, binary tree is generated where all branches have the same length. In the third step, the alignment itself is generated by assigning a nucleotide to each position in the alignment (or pair of positions when dealing with alignment columns which are base-paired in the corresponding RNA structure) from the respective equilibrium distribution, and then following the tree from its root to the leaves, assigning nucleotides to each node in the tree based on the transition matrices derived from the appropriate rate matrix. We use the same equilibrium distributions and rate matrices as Pfold [20] and SimulFold [32].

Structures selected from the RNA STRAND database were binned according to their sequence length (100–199, 200–299, etc. up to 900–999). For the tree length experiment, 10 structures were selected at random from each bin, and for each structure, alignments of 10 sequences were generated with total tree lengths of 0.5, 1, 2, 4, 8, and 16. The artificial data set for which the tree experiments were performed thus consists of 540 artificial alignments. For the alignment length experiment, 50 structures were selected at random from each bin. For each structure, we generated an alignment with 10 sequences and a total tree length of 4. The artificial data set for which the length experiments were performed consists therefore of 450 artificial alignments.

#### The *hok* data set

The *hok/sok* system in the R1 plasmid of *Escherichia coli* is responsible for maintaining the plasmid's presence through successive generations [87]. It comprises three genes: the *hok* (‘host-killing’) gene encoding a protein toxin, the *mok* (‘modulation of killing’) gene required for *hok* translation, and the *sok* (‘suppression of killing’) gene which blocks the translation of *mok*, thereby repressing *hok* [88]. The *hok/sok* system expresses two constitutively transcripts. One transcript, called the *hok* transcript, where the *hok* and *mok* reading frames overlap, and the other transcript corresponding to the *sok* RNA gene [89]. The *hok/sok* system stabilizes the plasmid by killing daughter cells that lack the plasmid after fission from the plasmid-containing parent. The way this happens is that daughter cells soon run out of *sok* transcripts to suppress *hok* translation because the constitutively-expressed *hok* transcript is more stable than the the also constitutively-expressed *sok* transcript which degrades quickly. The RNA structure of the *hok* transcript is key to this mechanism.

As the *hok* transcript emerges, it forms a metastable structure, which blocks the ribosomal binding sites for the *hok* and *mok* gene, thereby preventing premature ribosome loading. Once the whole transcript has been produced, the transcript adopts a stable inactive RNA structure. However, 3′ processing of this transcript allows the transcript to rearrange into the active structure, which is translationally active unless the *sok* transcript is bound to it. This metastable structure is likely to also guide the folding into the stable inactive structure (which comprises a ‘long-distance’ helix that pairs a region at the 5′ end of the transcript to a region near its 3′ end), preventing premature formation of the active structure.

A review of the *hok/sok* mechanism can be found in [88]. Several evolutionarily related toxin/antitoxin systems have been identified, and the alignment of their transcripts reveal covariation patterns consistent with each of these structures [90]. We choose the alignment from Gerdes *et al.* [88] which contains several more members of the *hok* family. It comprises a total of 9 sequences, has a length of 196 bp and a total tree length of 2.31. As this alignment provides only an outline of the helices, we manually derived the exact consensus structure from the observed evolutionary pattern. The Gerdes alignment does not cover the entire length of the *hok* transcript, but comprises the regions with most structural rearrangements.

#### The *trp*-attenuator data set

The *trp*-attenuator is a ribo-switch which regulates transcription of the *trp*-operon in *Escherichia coli* [91]. It is located in the leader peptide region of the *trp*-operon transcript, and can form three different helices. Two of the helices, the helix involving regions 1 and 2 (called the 1∶2 helix) and the 3∶4 helix (the numbering of regions is from 5′ to 3′), are mutually compatible, whereas the third 2∶3 helix is incompatible with either of the two other helices.

The formation of the 1∶2 helix during transcription causes the RNA-polymerase to pause. If a ribosome starts translation, it disrupts the 1∶2 helix as soon as it reaches region 1 of the transcript, thus freeing the RNA-polymerase. If tryptophan is limited, the ribosome will pause at the tryptophan codon in region 1, thereby allowing helix 2∶3 to form and simultaneously preventing the formation of the 3∶4 helix which serves as a terminator stem which ends transcription. This allows the *trp*-operon to be fully transcribed. If tryptophan is not limiting, however, the ribosome disrupts the 2∶3 helix, thereby allowing the 3∶4 helix to form and to terminate the transcription.

Several protein-mediated ribo-switches which regulate *trp*-operon activity have been identified in *Bacillus subtilis* [92]. The comparative analysis of *trp*-operons from several species of Actinobacteria show similar features to the *Escherichia coli trp*-attenuator [93]. The RNA structure detection program RNAlishapes can successfully identify all three helices from an alignment of several actinobacterial *trp*-attenuator sequences [94]. For our dataset, we choose the alignment proposed by Voss [94] which comprises 8 sequences, has a total length of 117 bp and a total tree length of 2.29.

### Performance evaluation

The performance of new prediction methods is best benchmarked by comparing the set of predicted to the set of known structure for an, ideally, large and diverse data set that has been carefully and completely annotated (the *test set*). If the prediction method depends on free parameters, these parameters should have been trained or manually derived from a *training set* which should have no overlap with the test test (and which has to be large and diverse enough to minimize the risk of parameter over-fitting). Typically, training and test sets are permuted several times in cross-evaluation experiments in order to show that both, the parameter training and the resulting performance are fairly independent of the particular choice of training and test set. This careful benchmarking is comparatively easy to accomplish for some applications, e.g. methods for RNA secondary structure prediction, but more difficult for others.

The conclusive benchmarking of computational methods for predicting kinetic folding pathways has, so far, been difficult. This is due to several reasons. First, detailed experimental results on folding kinetics, usually done via temperature- or pH-jump kinetic trapping procedures [95] or single-molecule ‘optical-tweezer’ manipulation [96], are only available for a small number of sequences which are typically quite short (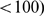 bp) and may, moreover, correspond to artificial sequences. Second, the assumptions made explicitly or implicitly by the prediction methods may not apply to the experimental setting. Third, there are no standard metrics for comparing experimental results with output from computational prediction methods (whose type of output varies greatly from method to method). Fourth, many computational methods (especially more heuristic ones [66], [97]) rely on a number of free parameters which require a dedicated test set in order to train them reliably and to avoid overlap with the test set. Consequently, most methods for predicting the RNA folding kinetics have been evaluated via a qualitative rather than quantitative comparison and only by considering a few chosen experimentally investigated sequences.

Transat has been devised to detect conserved RNA helices of statistical significance. Using Transat, we thus hope to not only detect the helices of the known functional RNA structure, but also new helices of functional importance which may be involved in defining the RNA's folding pathway *in vivo*.

#### Performance for detecting helices of known functional RNA secondary structures

Comparing the helices predicted by Transat to the helices of the known RNA secondary structures in our Rfam data set should allow us to get an estimation of Transat's performance. For the purposes of this evaluation, we assume that the structural annotation of this data set is not only correct, but also complete.

As we want to know how good Transat is at recovering helices of the known RNA secondary structure, we investigate the helix-specific performance in addition to the base-pair specific performance. We measure the performance in two ways: the sensitivity as function of the false positive rate (FPR) and the sensitivity as function of the positive predictive value (PPV).

As is customary, we define the sensitivity as 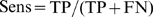, where TP is the number of true positives and FN is the number of false negatives. The false positive rate is defined as 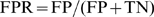, where FP is the number of false positives and TN is the number of true negatives. The positive predictive value is defined as 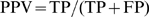. The sensitivity thus measures the fraction of known features that have been correctly predicted, whereas the positive predictive value corresponds to the fraction of predicted features that are correct. As we discard predicted helices with a p-value below a user-defined threshold of 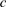, we classify base-pairs as defined in Table 1.

**Table 1:** Definitions regarding the base-pair-specific performance of Transat.

	base-pair is known structure	base-pair not in known structure
minimum p-value 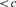	TP	FP
minimum p-value 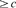	FN	TN

In order to quantify the performance of Transat, base-pairs are first classified into true positives (TP), true negatives (TN), false positives (FP) and false negatives (FN) according to the definitions above, where 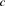 denotes the user-defined p-value threshold which is applied to the helices predicted by Transat. The minimum p-value of a predicted base-pair is defined as the minimum p-value of all predicted helices that contain this base-pair, i.e. a predicted base-pair inherits its statistical significance from the most statistically significant helix to which it belongs.

Transat's primary aim is to reliably detect statistically significant, conserved helices. It thus makes sense to investigate the helix-specific performance in some detail. We do this as before by investigating the sensitivity (Sens) as function of the false positive rate (FPR) as well as the sensitivity (Sens) as function of the positive predictive value (PPV). The above definitions for these three terms still apply, but the definitions for TP, TN, FP and FN have to be revised as we are now comparing helices rather than individual base-pairs. For a predicted helix to be considered a true positive (TP), we require 70% or more of its base-pairs to match known base-pairs. For a given p-value threshold of 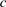, each helix is classified as defined in Table 2.

**Table 2:** Definitions regarding the helix-specific performance of Transat.

	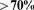 known base-pairs	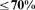 known base-pairs
p-value 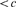	TP	FP
p-value 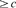	FN	TN

In order to quantify the performance of Transat for entire helices, helices are first classified into true positives (TP), true negatives (TN), false positives (FP) and false negatives (FN) according to the definitions above, where 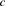 denotes the user-defined p-value threshold which is applied to the helices predicted by Transat.

**Figure 4:**
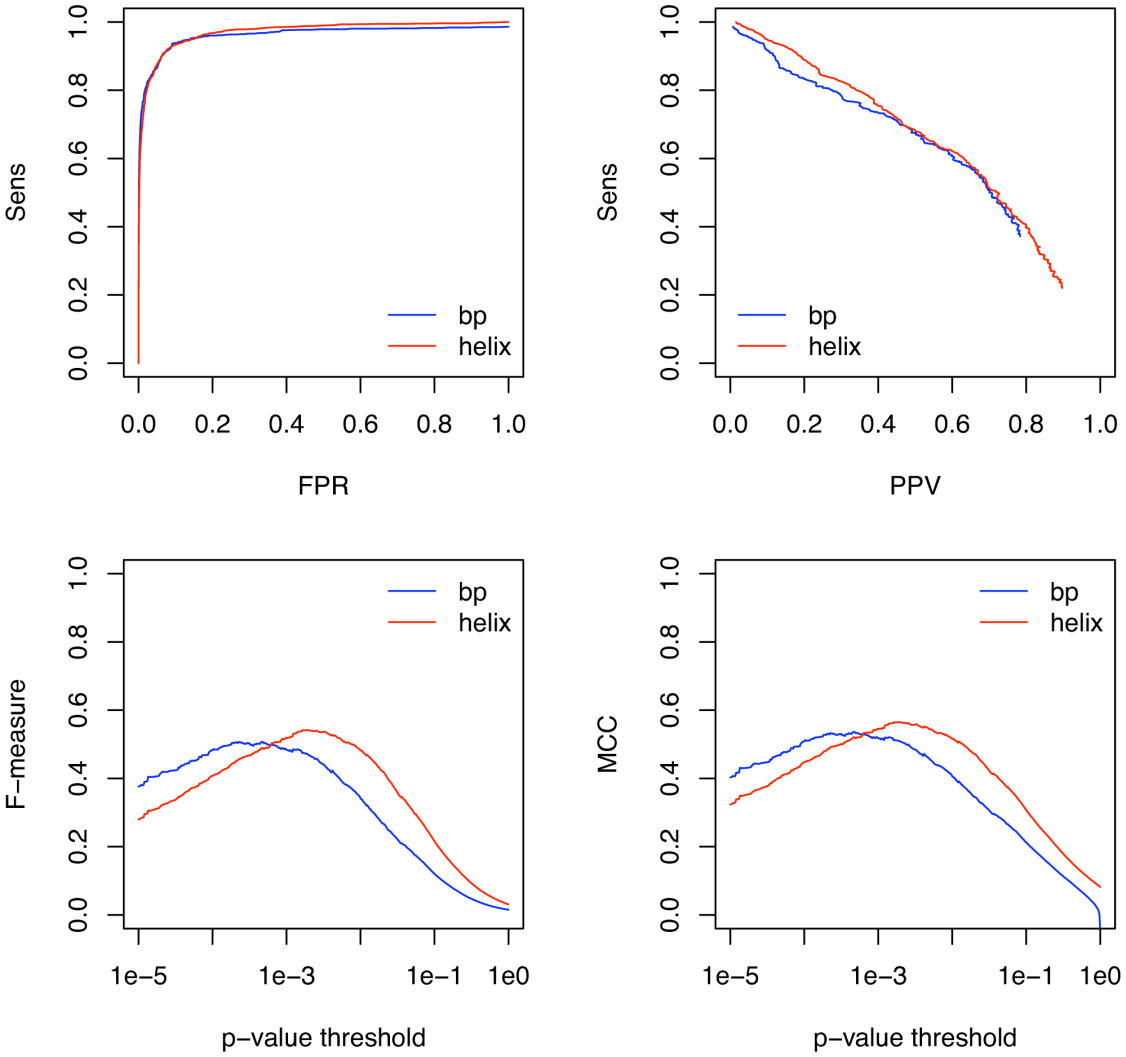
Performance of Transat for detecting the known base-pairs (bp) and helices (helix) of the Rfam data set. The top left figure shows the sensitivity as function of the false positive rate (FPR) and the top right figure the sensitivity (Sens) as function of the positive predictive value (PPV). The bottom left figure shows the F-measure and the bottom right figure the MCC as function of the p-value threshold, see the text for the definitions of the F-measure and the MCC. Note that each data point in the figures above corresponds to the respective performance measure averaged over the entire Rfam data set for a particular p-value threshold (along the x-axis).

As Figure 4 shows, Transat has almost the same performance for helices as for individual base-pairs. For many applications, one wants to maximize the sensitivity and the PPV at the same time. A good way to visualize how both performance measures vary with the p-value threshold is thus to plot the F-measure as function of the p-value threshold as shown in Figure 4. The F-measure or F-score is defined as the harmonic mean of the sensitivity and the PPV, i.e. 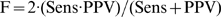. The helix specific F-measure reaches its maximum value of 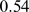 for a p-value threshold of 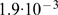, whereas the base-pair specific F-measure peaks at a p-value threshold of 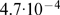 with an F-measure of 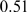. Another measure which combines several performance indicators into one is the so-called Mathews correlation coefficient (MCC) which is defined as 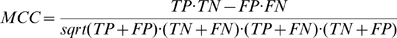. It is similar, but not identical to the F-measure, see Figure 4. We expect the observed sensitivity to be an indicator of Transat's true performance. As we do not know for sure, however, whether or not the known structural annotation is complete, i.e. if all functional helices have been annotated, the measured positive predictive value can be viewed as a lower boundary to the true performance of Transat.

#### Dependence of the performance on the alignment length and the total tree length

**Figure 5:**
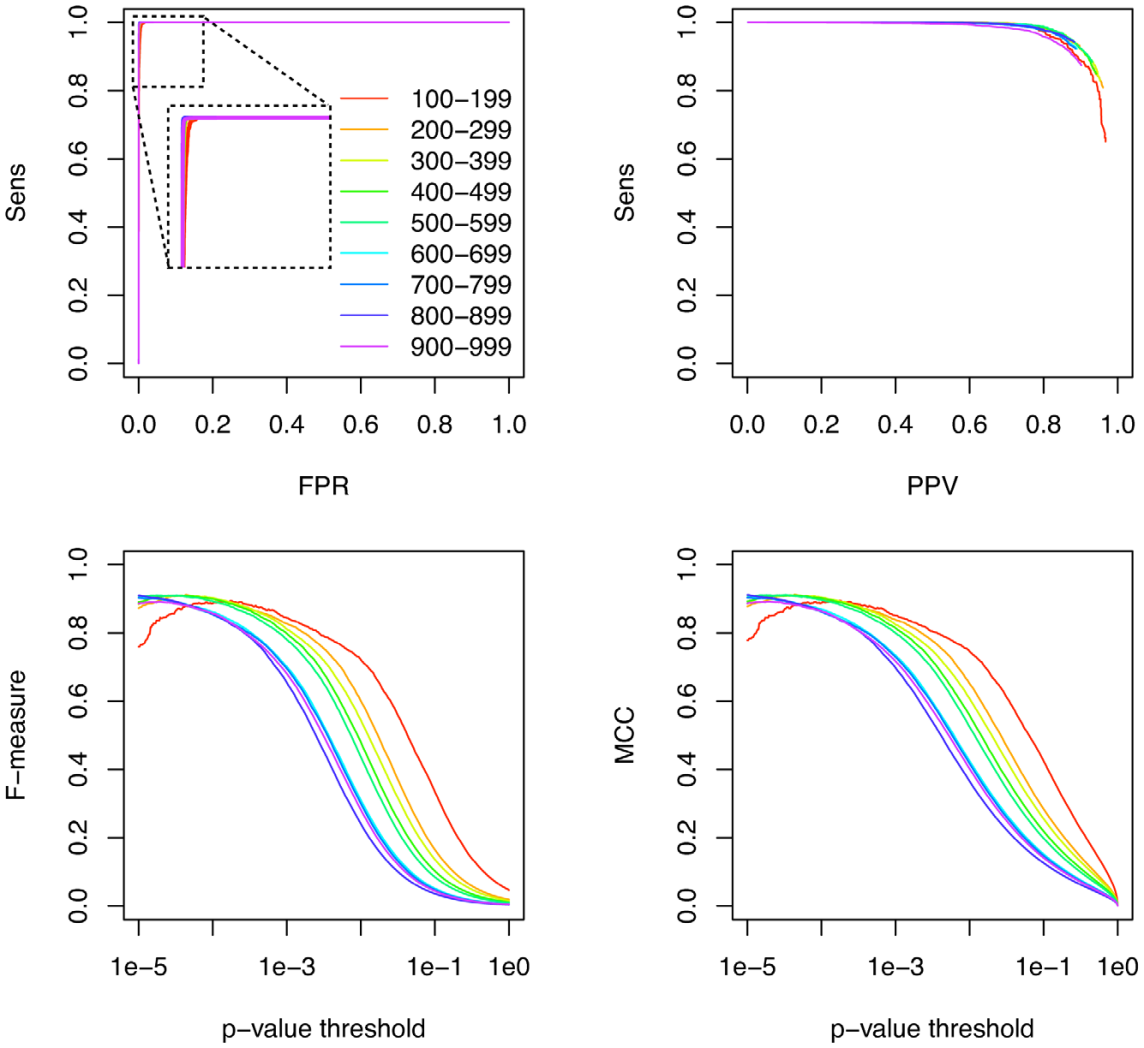
Performance of Transat for predicting the known helices of the artificial data set as function of the alignment length. The top left figures shows the sensitivity (Sens) as function of the false positive rate (FPR) for different alignment lengths. The colors indicate the length of the alignment in nucleotides ranging from 100 to 999 nucleotides. The top right figures shows the sensitivity as function of the positive predictive value (PPV) for different alignment lengths. The bottom left figures shows the F-measure and the bottom right figure the MCC as function of the p-value threshold, see the text for the definitions of the F-measure and the MCC. All figures use the same coloring scheme as the top left figure.

The performance of methods that predict a kinetic folding pathway is known to strongly depend on the length of the input sequence. In order to systematically investigate to which extent the performance of Transat depends on the alignment length, we investigate the predictions for the artificial data set. As Figure 5 shows, the sensitivity as function of the false positive rate shows no perceptible dependence on the length of the alignment, whereas the sensitivity as function of the PPV decreases slightly as the length of the alignment increases.

**Figure 6:**
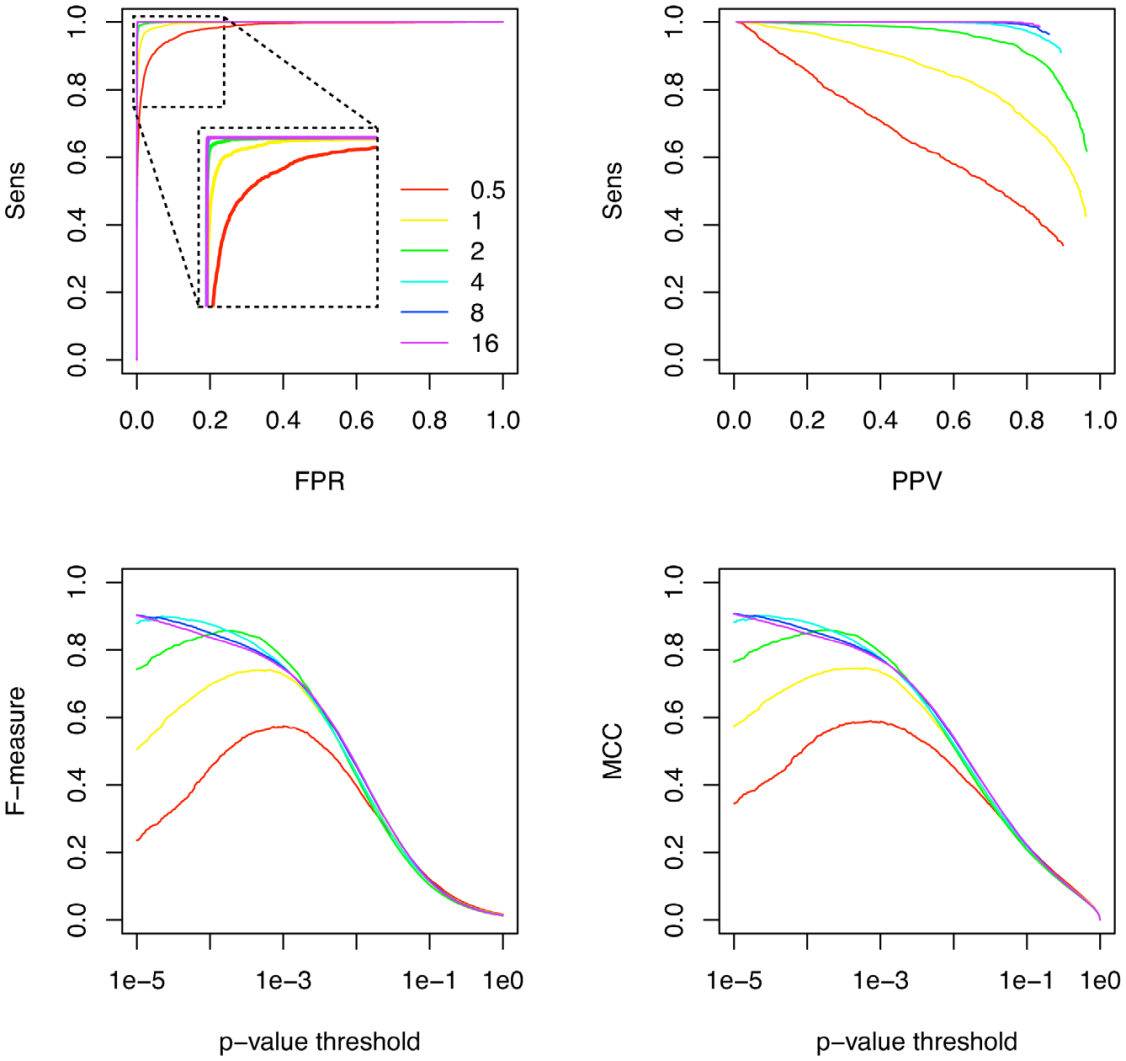
Performance of Transat for predicting the known helices of the artificial data set for different total tree lengths. The top left figures shows the sensitivity (Sens) as function of the false positive rate (FPR) for different tree lengths. The colors indicate the total length of the maximum-likelihood trees that were derived for the alignments of the artificial data set. They range from 0.5 to 16. The top right figures shows the sensitivity as function of the positive predictive value (PPV). The bottom left figures shows the F-measure and the bottom right figure the MCC as function of the p-value threshold, see the text for the definitions of the F-measure and the MCC. All figures use the same coloring scheme as the top left figure.

Transat is a comparative method, whereas all existing methods for predicting kinetic folding pathways take a single RNA sequence as input. It is well known that the performance of comparative RNA secondary structure prediction methods depends on the number of sequences in the alignment, see e.g. [19], or, more precisely, on the total tree length of the sequences in the input alignment, see e.g. [22]. In order to investigate how Transat's performance varies with the total tree length, we investigate the predictions for the artificial data set. We calculate the maximum-likelihood (ML) tree as the reference tree for all alignments in our artificial data set. As Figure 6 shows, the performance increases with the total tree length. This dependence is more pronounced for the sensitivity as function of the PPV than the sensitivity as function of the false positive rate.

When comparing the performance plots for the artificial data set to those for the Rfam data set shown in Figure 4, it is clear that the performance for the artificial data set is superior. This is mainly due to two reasons. First, due to the way the artificial data set was constructed, it does not contain any conserved structural features that are not already part of the structural annotation, whereas we cannot say for sure whether or not the structural annotation of the Rfam data set is really complete. This affects in particular the sensitivity as function of the positive predictive value. Second, (again due to the way the artificial data set was generated) its alignments do not contain any alignment errors and no structural variation between sequences of the same alignment, whereas the alignments of the Rfam data set may be affected by both types of complications. The performance evaluation for the artificial data set thus presents only an idealized view of the program's true performance, but has the advantage of allowing us to study the influence of the alignment length and the total tree length in great detail and without having to take additional complications into account.

When comparing the effect of the total tree length on the performance to the effect that the alignment length has, it is clear that evolutionary diversity, i.e. the total tree length, has a much greater influence on the performance than the alignment length. This can be understood by the way that Transat generates predictions. Transat detects conserved helices by identifying pairs of co-varying alignment columns. We expect the amount of co-variation to strongly depend on the total tree length. If the sequences in the input alignment look very similar, i.e. if they are closely related and if the corresponding total tree length is small, the amount of co-variation will be significantly smaller than if the sequences are evolutionarily more distantly related, i.e. if the total tree length is large. If the sequences are only very distantly related, we expect structural variation to occur between the sequences, e.g. helices that have been conserved in some sequences, but not in others. This effect cannot be observed in our artificial data set, but has been shown to exist in some biological data sets, see e.g. [22].

The number of possible bi-secondary RNA structures, i.e. RNA structures that can be viewed as combination of at most two secondary structure without pseudo-knots, grows exponentially with the sequence length [98]. For a non-comparative method that predicts structure elements such as helices, we thus expect the PPV to significantly decrease with the sequence length. For a comparative method, however, we expect this effect to be less pronounced because there is no reason to expect the number of structural features *that are supported by co-variation* to also increase quadratically with the alignment length. This is, in our view, the main reason why comparative methods tend to outperform non-comparative methods. The alignment length has, however, an effect on the p-values that are estimated for the detected helices. This, more minor, effect is shown in Figure 5.

#### Transat predictions for the *hok* and *trp*-attenuator data sets

The *hok* and *trp*-attenuator data sets allow us to evaluate the performance of Transat for sequences, where more than a single functional RNA secondary structure is known.

**Figure 7:**
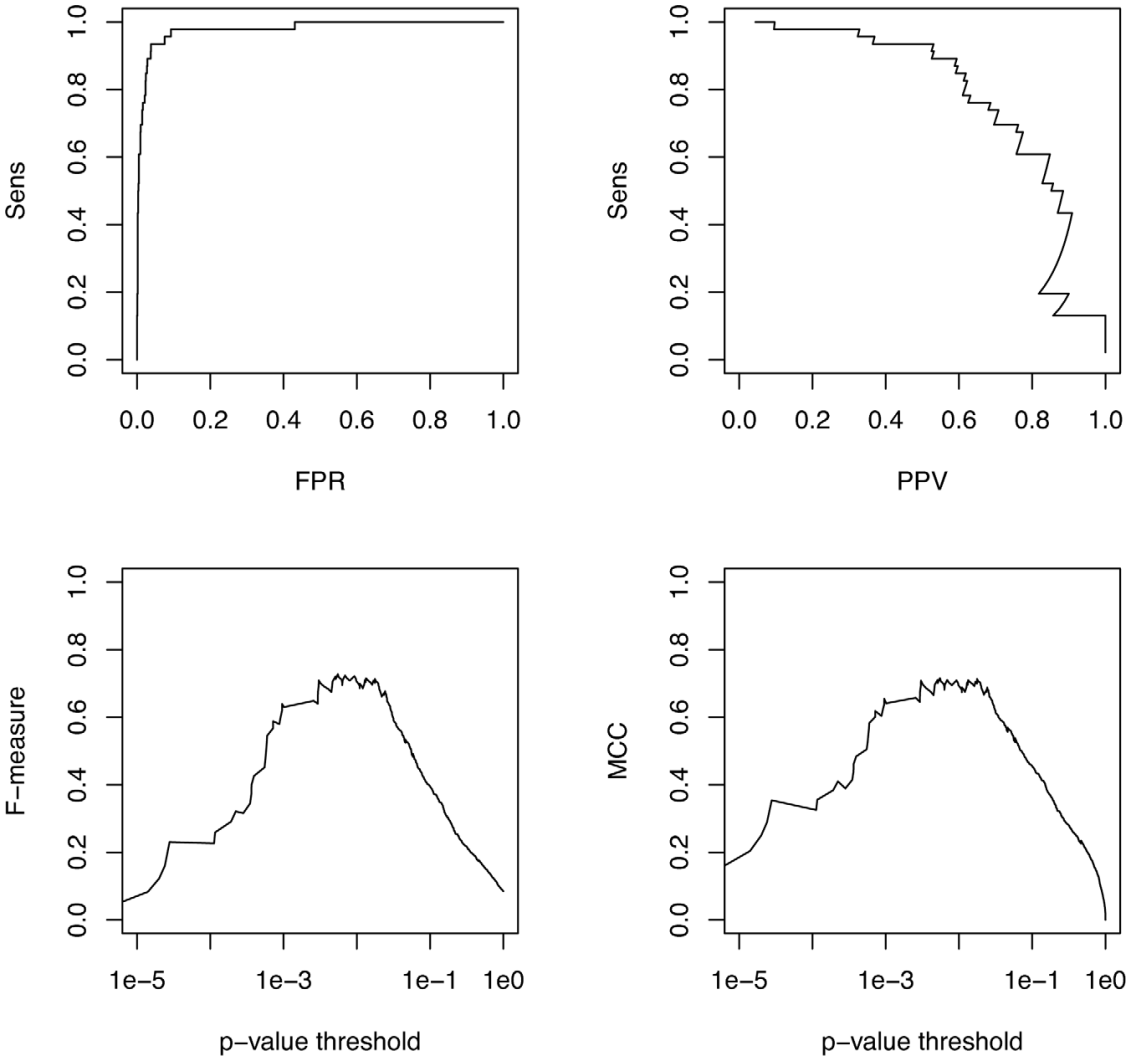
Performance of Transat for detecting the known helices of the *hok* data set. The top left figures shows the sensitivity as function of the false positive rate (FPR) and the top right figure the sensitivity as function of the positive predictive value (PPV). The bottom left figure shows the F-measure and the bottom right figure the MCC as function of the p-value threshold, see the text for the definitions of the F-measure and the MCC.

**Figure 8:**
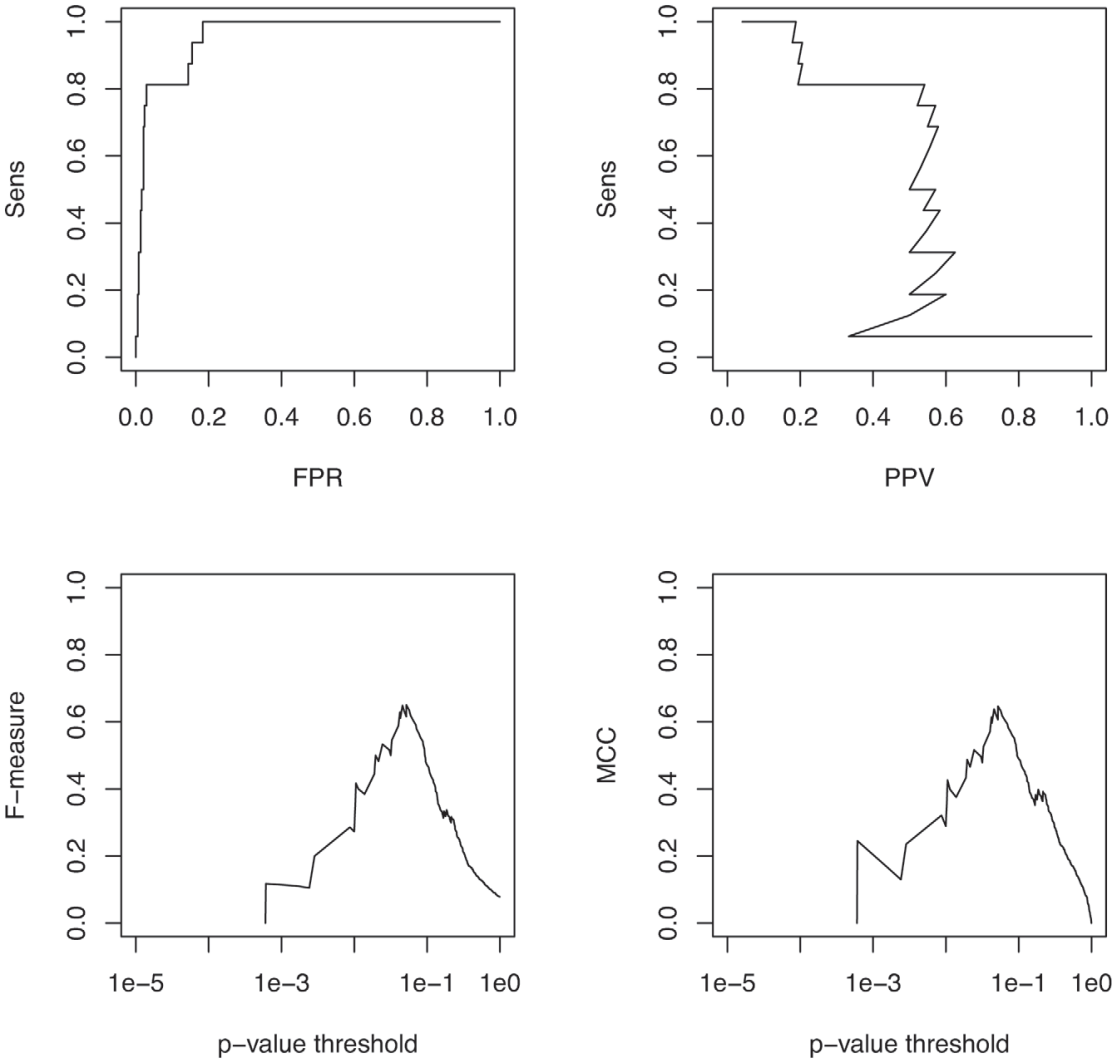
Performance of Transat for detecting the known helices of the *trp*-attenuator data set. The top left figures shows the sensitivity as function of the false positive rate (FPR) and the top right figure the sensitivity as function of the positive predictive value (PPV). The bottom left figure shows the F-measure and the bottom right figure the MCC as function of the p-value threshold, see the text for the definitions of the F-measure and the MCC.

The performance of Transat for the *hok* data set is shown in Figure 7, those for the *trp*-attenuator data set in Figure 8. These figures show how the performance varies as the p-value threshold value is changed. The F-measure for the *hok* data set peaks with a value of 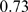 for a p-value threshold of 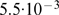, whereas the F-measure for the *trp*-attenuator data set peaks with a value of 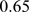 for a p-value threshold of 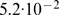. Transat can thus successfully detect the alternative structure of the *hok* data set, whereas the performance for the *trp*-attenuator data set is not as high. The peak performance values for these two data sets where known transient helices exist is, however, still significantly larger than the peak performance for the Rfam data set (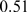 for base-pairs and 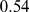 for helices).

**Figure 9:**
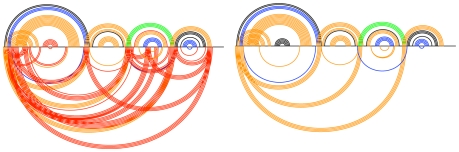
Conserved helices predicted by Transat for the *hok* data set for different p-value threshold values (left 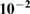, right 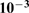). The x-axis represents the *hok* alignment. Each arc corresponds to a base-pair between the respective positions in the alignment. Arcs above the x-axis correspond to known base-pairs, whereas arcs below correspond to new base-pairs predicted by Transat, i.e. they correspond to base-pairs that do not involve the same pair of nucleotide positions as any base-pair in the known structure(s). Base-pairs predicted by Transat have non-black colours which indicate their reliability as estimated by Transat (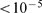 green, 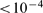 blue, 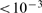 orange and 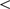 (p-value threshold) red). They can either be found above the x-axis, if they agree with a pair in the reference structure(s), or below, if they are new. Transat predicts most helices of the known structure as well as three statistically significant conserved helices which may guide the structure formation.

**Figure 10:**
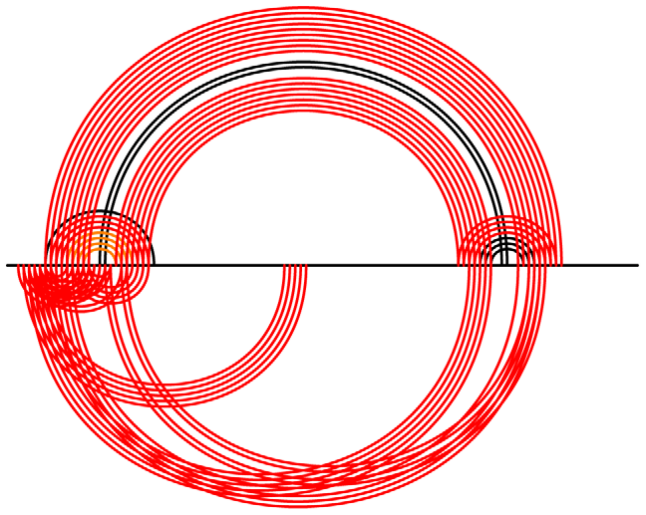
Conserved helices predicted by Transat for the *trp*-attenuator data set as function for a p-value threshold of 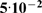. The x-axis represents the *trp*-attenuator alignment, see the text or the caption of Figure 9 for more information on arc-plots. Transat predicts almost all base-pairs of the known structure correctly as well as several equally significant conserved helices which may guide the formation of the known structure.

A more intuitive way of visualizing the Transat predictions is to plot the predicted helices for a given p-value threshold as shown in Figure 9 and Figure 10. These so-called arc-plots show the known structure as well as the predictions made by Transat. The x-axis symbolizes the alignment that was used as input to Transat. Each arc corresponds to a base-pair between the respective positions in the alignment. Arcs drawn above the x-axis correspond to base-pairs that are known to exist in the known RNA secondary structure (black) and that Transat predicts correctly (non-black colors), whereas arcs drawn below the x-axis correspond to new base-pairs predicted by Transat. The top arcs therefore visualize the sensitivity, whereas the bottom arcs visualize the positive predictive value of Transat. The colors of the individual arcs indicate the minimum p-value of the respective base-pair as estimated by Transat (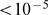 green, 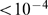 blue, 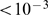 orange and 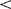 (p-value threshold) red). As defined earlier, the minimum p-value of a predicted base-pair corresponds to the minimum p-value of all predicted helices that contain this base-pair.

#### Revisiting the Transat predictions for the Rfam data set

The Rfam data set allows us to evaluate Transat's performance for detecting the known references structures as presented above. For this, we assume the structural annotation of the Rfam data set to be both, correct and complete. Any Rfam alignment, however, only corresponds to a single functional RNA secondary structure in the Rfam data base and does not contain information on alternative functional RNA secondary structure or conserved transient helices. It may thus be possible to detect additional, evolutionarily conserved structural features with Transat that are currently not part of the structural annotation. For this, we studied several Rfam families in greater detail.

**Figure 11:**
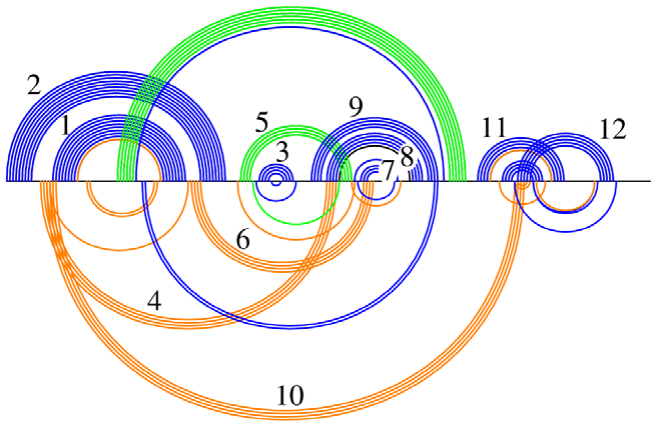
Known RNA secondary structure and Transat predictions for the Cripavirus internal ribosomal entry site (IRES), RF00458, for a p-value threshold of 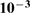. Transat predicts the helices of the pseudo-knotted known structure correctly and also predicts several transient helices which suggest a co-transcriptional folding pathway (see numbering of helices above). All predicted transient helices (helices 4, 6 and 10) are mutually incompatible with a helix of the known RNA structure. Helix 4 may yield to helix 8, helix 6 to helix 7 and helix 10 to helix 12. The transient helices may thereby guide the formation of the known functional RNA structure.

Figure 11 shows the Transat predictions for the Cripavirus internal ribosomal entry site (IRES). Transat not only detects the helices of the known, pseudo-knotted RNA secondary structure, but also predicts several transient helices of lower statistical significance which may be involved in defining the RNA's co-transcriptional folding pathway. As shown in Figure 11, it is fairly easy to manually bring all helices into an order in which they may appear in the co-transcriptional folding pathway (see helices 1 to 12).

**Figure 12:**
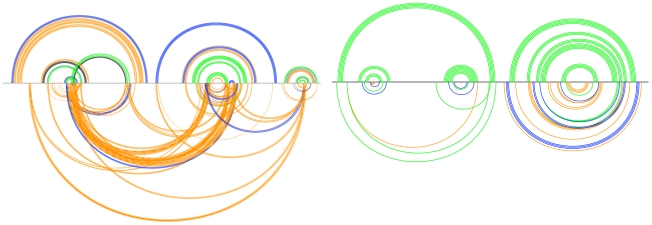
Known RNA secondary structure and Transat predictions for telomerase RNA for vertebrates (left, RF00024) and ciliates (right, RF00025) for a p-value threshold of 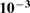. The Transat predictions indicate that the co-transcriptional folding of the vertebrate sequences may involve large-range structural rearrangements, whereas the two hair-pins of the known ciliate structure are predicted to form independently. Note that Transat correctly captures the known pseudo-knotted structure of the vertebrate telomerase RNA (left). See the text or the caption of Figure 9 for more information on arc-plots.

Figure 12 shows the results for telomerase RNA, once for vertebrate sequences and once for ciliate sequences. Note that Transat correctly captures the pseudo-knotted structure of the vertebrate telomerase RNA. As the Transat predictions show, the vertebrate data set contains evidence for a conserved helix of high statistical significance (see blue helix with p-value 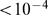) linking the first two hairpin-like structures which would involve large-range structural rearrangements, whereas the ciliate sequences do not support such a helix. Similar to the predictions shown in Figure 11, transient helices predicted by Transat are often mutually incompatible. This arrangement enforces an ordered way of rearranging the emerging RNA secondary structure while at the same time minimizing the sequence space occupied by these transient helices.

**Figure 13:**
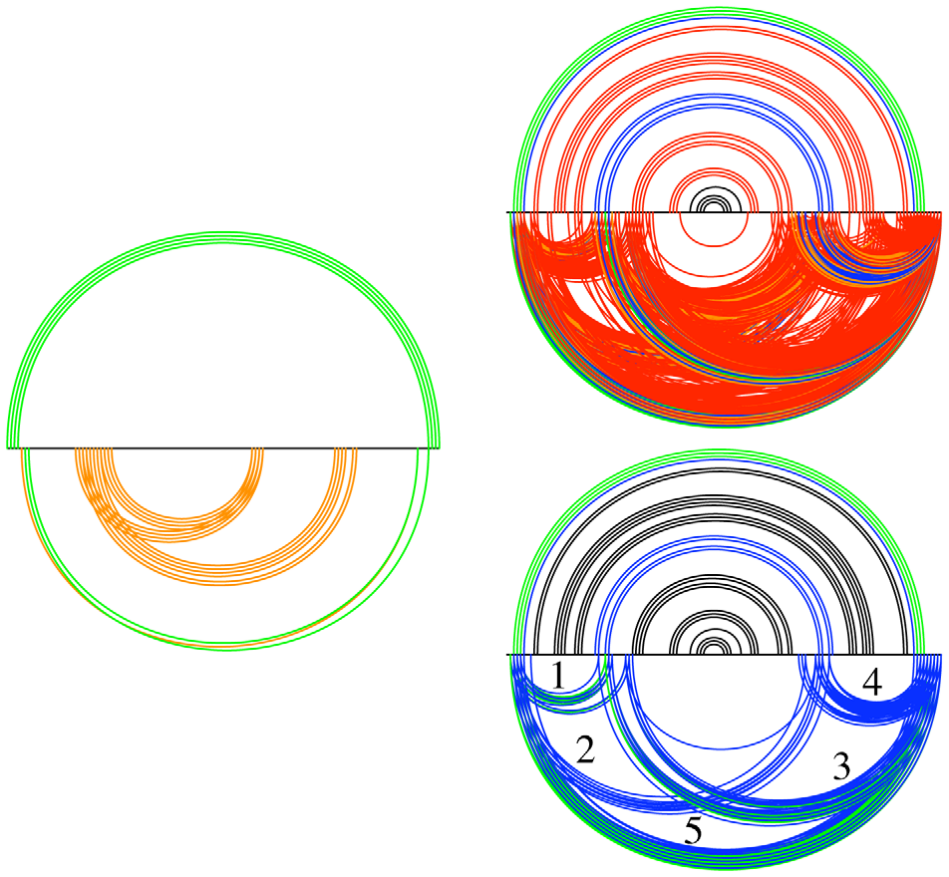
Known RNA secondary structure and Transat predictions for two hairpin-like known structures, the small nucleolar RNA snR76 for a p-value threshold of 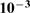 (left, RF01209) and the bacterial signal recognition particle RNA (right, RF00169) for a p-value threshold of 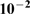 (right, top) and 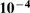 (right, bottom). The Transat predictions for both RNA families indicate several, mutually incompatible transient helices. In case of the bacterial signal recognition particle, the transient helices (right, bottom, numbered 1–5) are mutually incompatible with the base-pairs of the known structure. The hairpin-like structure of the small nucleolar RNA snR76 seems to fold in one go, whereas the formation of the hairpin-like structure of the bacterial signal recognition particle RNA may first involve the formation of helix 1 which is later replaced by the known hairpin-like structure as the RNA sequence gets further transcribed. Helices 2 to 5 are predicted as statistically more significant (p-values 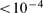) than the helices of the known hairpin-like structure. They are mutually exclusive and may correspond to alternative structural confirmations for this sequence. See the text or the caption of Figure 9 for more information on arc-plots.

Figure 13 shows the Transat predictions for two hairpin-like known structures which suggest that they fold in different ways. Both alignments are roughly of the same length (RF01209 121 bp, RF00169 129 bp). The hairpin-like structure of the small nucleolar RNA snR76 seems to fold in one go, whereas the formation of the hairpin-like structure of the bacterial signal recognition particle RNA may first involve the formation of helix 1 which is later replaced by the known hairpin-like structure as the RNA sequence gets further transcribed. Helices 2 to 5 are predicted as statistically more significant (p-values 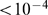) than the helices of the known hairpin-like structure. They are mutually exclusive and may correspond to alternative structural confirmations for this sequence.

**Figure 14:**
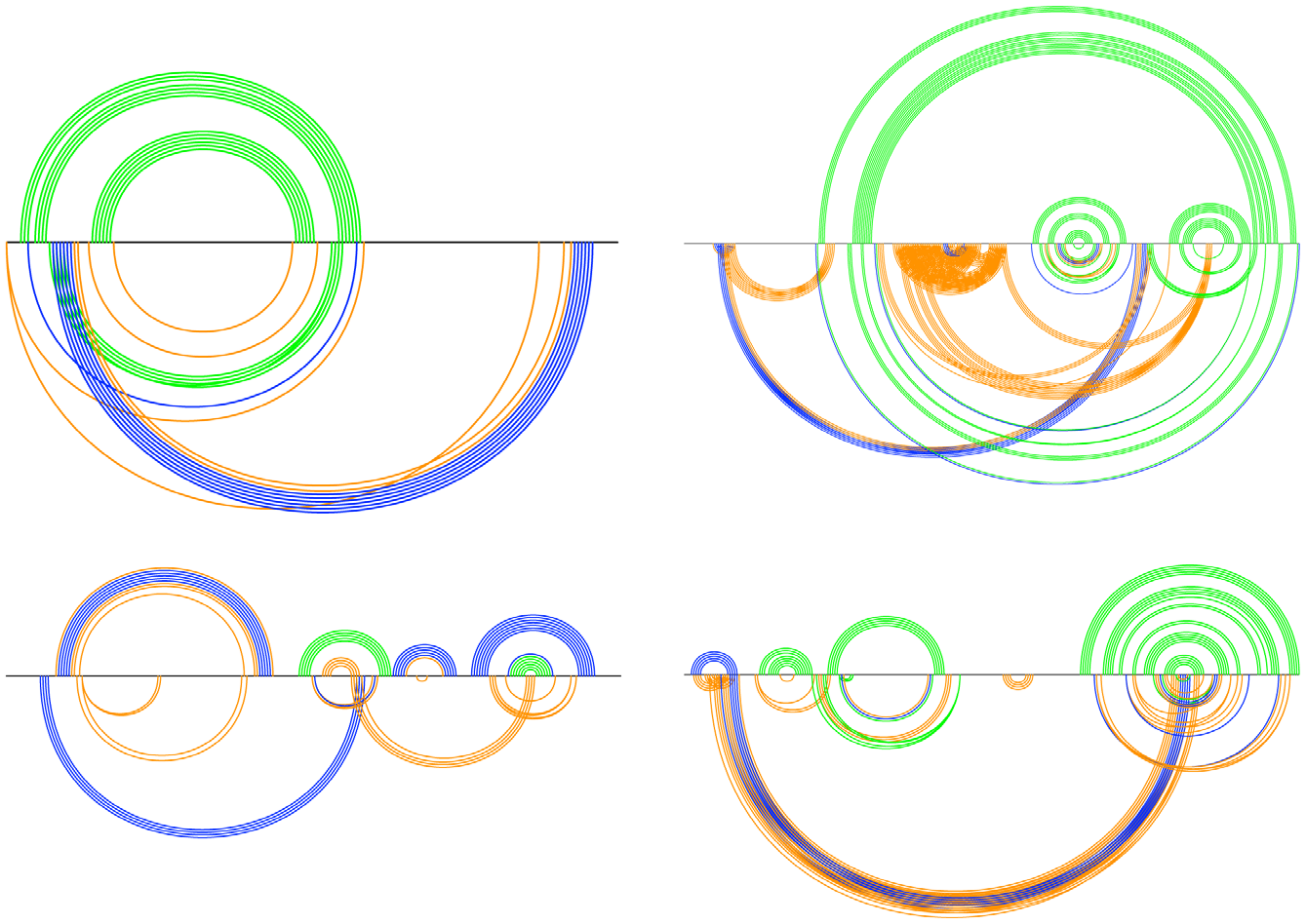
For several Rfam families, the Transat predictions propose a pseudo-knotted configuration, see the S-adenosyl-L-homocysteine ribo-switch (left, top, RF01057), the glmS glucosamine-6-phosphate activated ribozyme (left, bottom, RF00234), the small nucleolar RNA U3 (right, top, RF00012) and the U12 minor spliceosomal RNA (right, bottom, RF00007). For a p-value threshold of 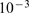, Transat predicts the helices of the known structures correctly and also provides strong statistical evidence (p-value 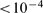) for additional helices that would render the known secondary structure pseudo-knotted, see the blue bottom-arcs for all four RNA families. Note that for the U12 minor spliceosomal RNA (right, bottom), the newly predicted helix is in competition with the most 5′ helix that is part of the known RNA secondary structure. See the text or the caption of Figure 9 for more information on arc-plots.

The four Rfam alignments presented in Figure 14 show that Transat provides strong evidence that a pseudo-knotted configuration is part of the co-transcriptional folding pathway or the annotated, functional RNA secondary structure. The latter is likely given that most RNA secondary structure prediction program ignore pseudo-knots and that human annotators could have easily missed the new helix whose two halves are far apart.

**Figure 15:**
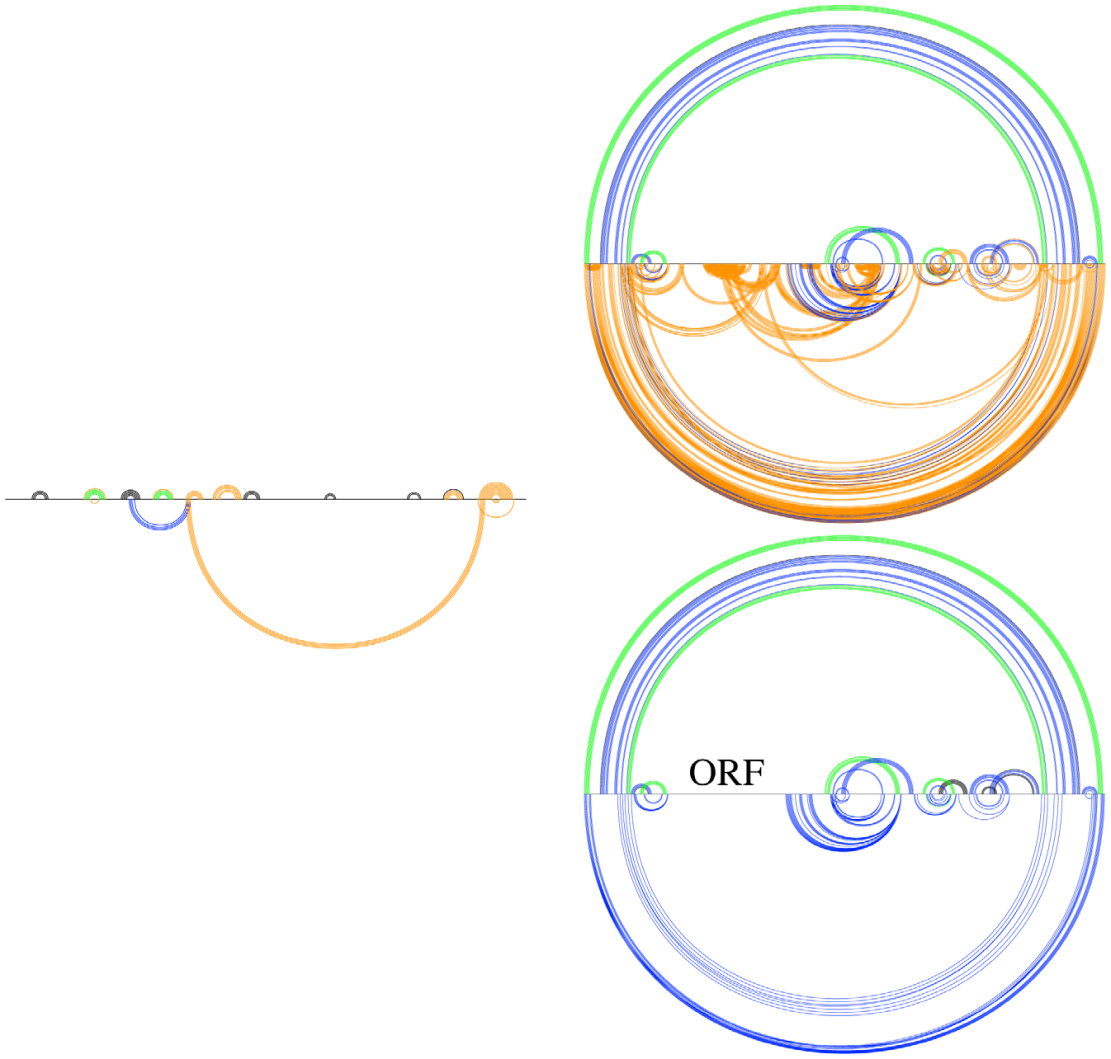
For some Rfam families, Transat highlights regions which are devoid of transient structures, thereby indicating regions of the RNA sequence which may be bound by other molecules early on in the folding process. Shown here are two examples, the CsrB/RsmB RNA family (left, RF00018) and the bacterial tmRNA (right, RF00023) for a p-value threshold value of 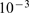 (left and right, top) and 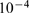 (right, bottom). The CsrB/RsmB RNA is known to be bound by multiple copies of the CsrA protein. The RNA's known structure comprises only short range helices and Transat predicts only two transient structures for the entire 392 bp long alignment. Both findings support the hypothesis that protein binding occurs early during the folding of this RNA. The helices of the pseudo-knotted known structure for the bacterial tmRNA are correctly predicted by Transat for a p-value threshold of 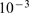 (right, top). Transat predicts several additional helices, but the region of the tmRNA sequences that contains the reading frame which ends in a translation stop signal is devoid of statistically significant transient helices (right, bottom) supporting the hypothesis that the sequence in that region of the has been chosen to remain single-stranded and readily accessible. See the text or the caption of Figure 9 for more information on arc-plots.

One motivation for devising Transat was to develop a method that does not require a detailed modeling of the *in vivo* environment, in particular of molecules binding to the RNA sequence which may involve the resulting folding pathway. As the two examples for alignments of length 392 bp (RF00018) and 655 bp (RF00023) in Figure 15 show, Transat is capable of highlighting sequence regions which are likely to be bound by other molecules or which are required to be single-stranded for proper functioning. These regions correspond to sub-sequences where Transat detects no conserved helices of statistical significance.

#### Comparison of the Transat predictions to the Boltzmann ensemble of RNA structures in thermodynamic equilibrium

**Figure 16:**
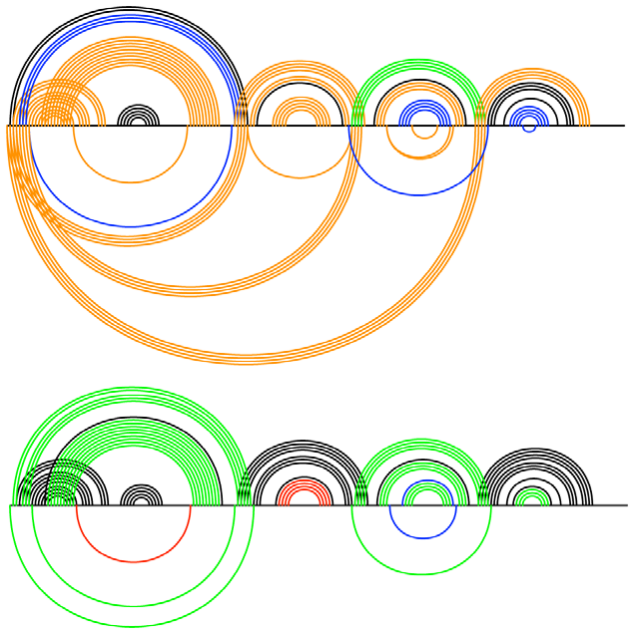
Comparison of Transat (top figure) and RNAalifold P (bottom figure) for the *hok* data set. In each figure, the x-axis represents the *hok* alignment. Each arc corresponds to a base-pair between the respective positions in the alignment. Arcs above the x-axis correspond to known base-pairs, whereas arcs below correspond to new base-pairs predicted by the respective program, i.e. they correspond to base-pairs that do not involve the same pair of nucleotide positions as any base-pair in the known structure(s). In the top figure, base-pairs predicted by Transat have non-black colours which indicate their reliability as estimated by Transat (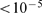 green, 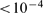 blue, 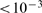 orange) using a p-value threshold of 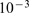. These base pairs can either be found above the x-axis, if they agree with a pair in the reference structure(s), or below, if they are new. In the bottom figure, base-pairs predicted by RNAalifold P have non-black colours which indicate their base-pairing probability in the Boltzmann ensemble of pseudo-knot free RNA secondary structures that we would expect in thermodynamic equilibrium (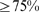 green, 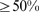 blue, 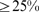 orange and 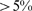 red) using a pairing probability threshold of 5%. These base-pairs can either be found above the x-axis, if they agree with a pair in the reference structure(s), or below, if they are new. Transat predicts most helices of the known structures as well as three statistically significant conserved helices which may guide the structure formation, whereas RNAalifold P predicts only part of the known structures and contributes only a few novel base-pairs which extend a known helix by one or two base-pairs on either side, see also Figure 9.

It is interesting to investigate if the helices predicted by Transat are similar to the structural features that would be present in thermodynamic equilibrium. For this, we use the program RNAalifold [17] with the “-p” option in order to calculate the probabilities of individual base-pairs in the Boltzmann distribution of all possible (pseudo-knot free) RNA secondary structures that are expected to be present in thermodynamic equilibrium. As Transat, RNAalifold P takes as input a fixed multiple-sequence alignment. We then compare these probabilities (i.e. those estimated by RNAalifold P for individual base-pairs) to the p-values assigned by Transat to individual helices. Using the same strategy as we used for Transat for deriving a sensible p-value threshold, we derive the threshold value which maximizes the performance for the Rfam data set in terms of F-measure by comparing all base-pairs predicted by RNAalifold P to those of the known reference structures. This results in a probability threshold of 5% which we use to compare the predictions by RNAalifold P to those of Transat, see Figure 16 to [Fig pcbi-1000823-g017] [Fig pcbi-1000823-g018] [Fig pcbi-1000823-g019] Figure 20 as well as Figures 3 to 10 in Text S1 (the file with supplementary information).

**Figure 17 pcbi-1000823-g017:**
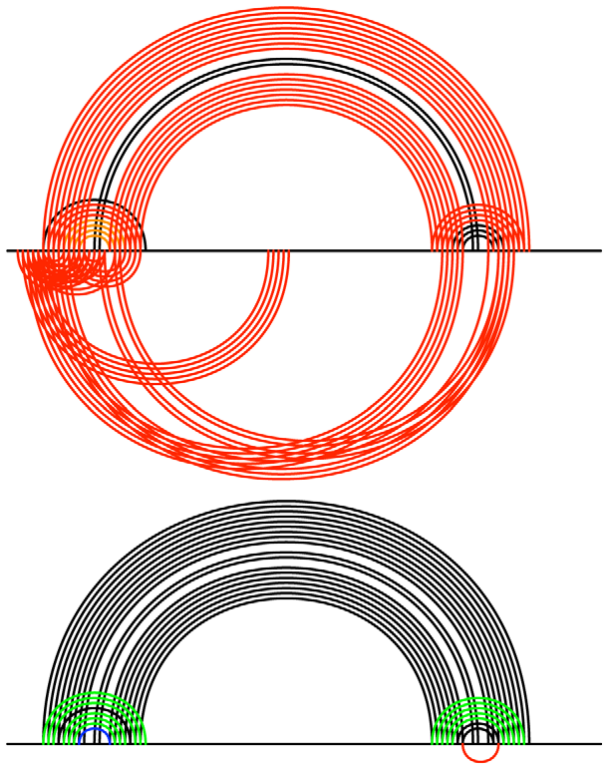
Comparison of Transat (top figure) and RNAalifold P (bottom figure) for the *trp*-attenuator data set. In the top figure showing the Transat predictions, base-pairs predicted by Transat have non-black colours which indicate their reliability as estimated by Transat (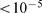 green, 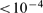 blue, 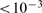 orange and 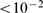 red) using a p-value threshold of 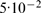. The bottom figure shows the RNAalifold P predictions, see the caption of Figure 16 for more information on arc-plots. Transat predicts all helices of the known structures and several new helices, albeit with relatively high p-values between 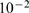 and 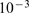), whereas RNAalifold P captures only two of the helices and proposes an single new base-pair which extends of the known helices, see also Figure 10.

**Figure 18 pcbi-1000823-g018:**
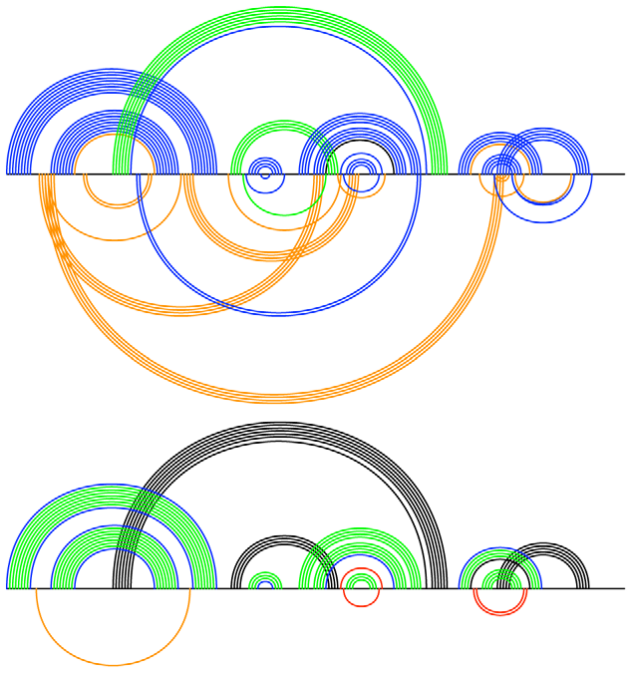
Comparison of Transat (top figure) and RNAalifold P (bottom figure) for the Cripavirus internal ribosomal entry site (IRES), RF00458. Transat predicts the helices of the pseudo-knotted known structure correctly and also predicts several new helices, whereas RNAalifold P captures only part of the known structure and predicts three new base-pairs which extend three known helices, see also Figure 10. Please refer to the caption of Figure 16 for more information on these arc-plots.

**Figure 19 pcbi-1000823-g019:**
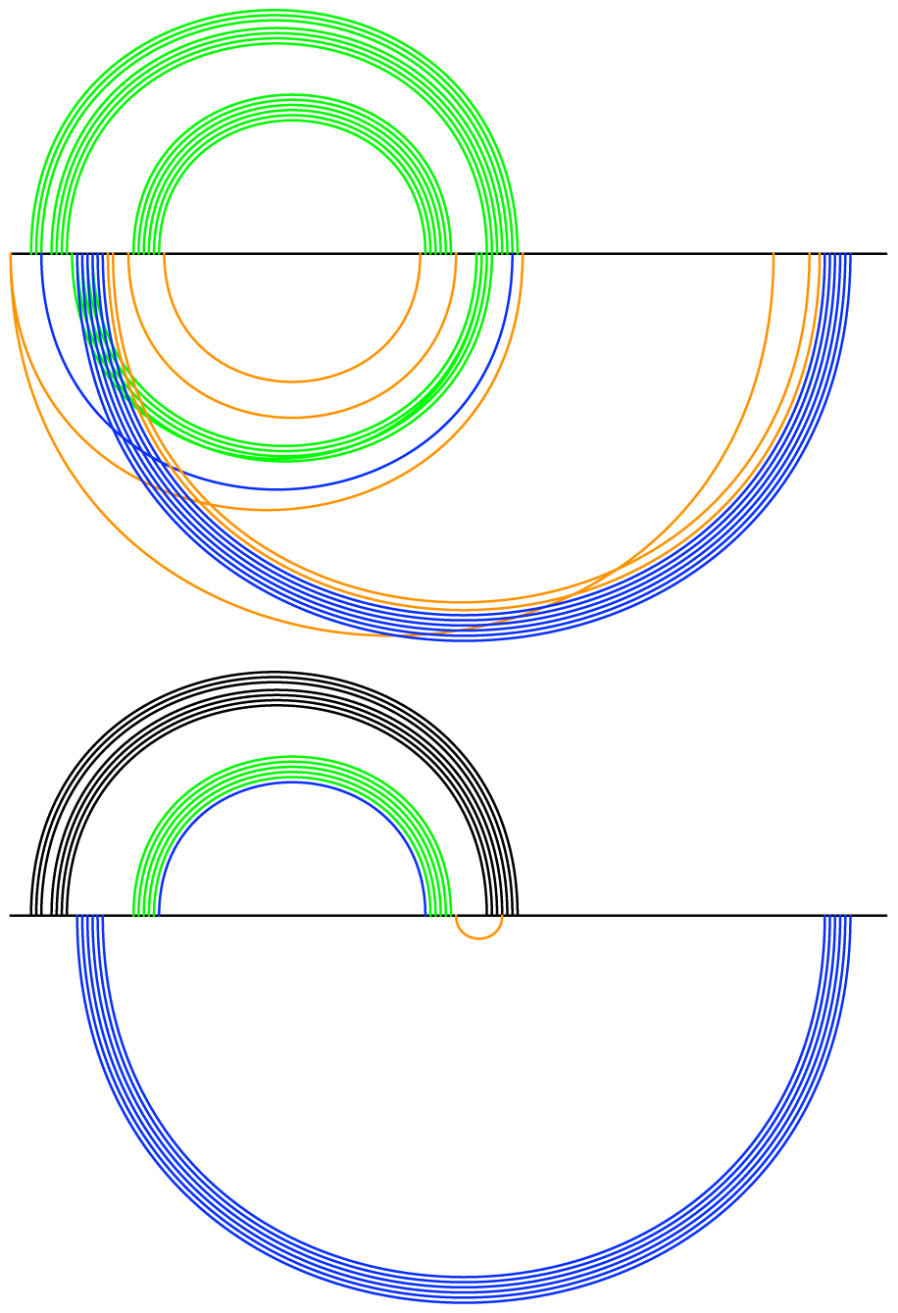
Comparison of Transat (top figure) and RNAalifold P (bottom figure) for the S-adenosyl-L-homocysteine ribo-switch, RF01057. Transat predicts the helices of the known structures correctly and also provides strong statistical evidence (p-value 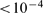) for additional helices that would render the known secondary structure pseudo-knotted, see the blue bottom-arcs. RNAalifold P predicts only part of the known structure correctly, but proposes a similar new helix, see also Figure 14. Please refer to the caption of Figure 16 for more information on arc-plots.

**Figure 20:**
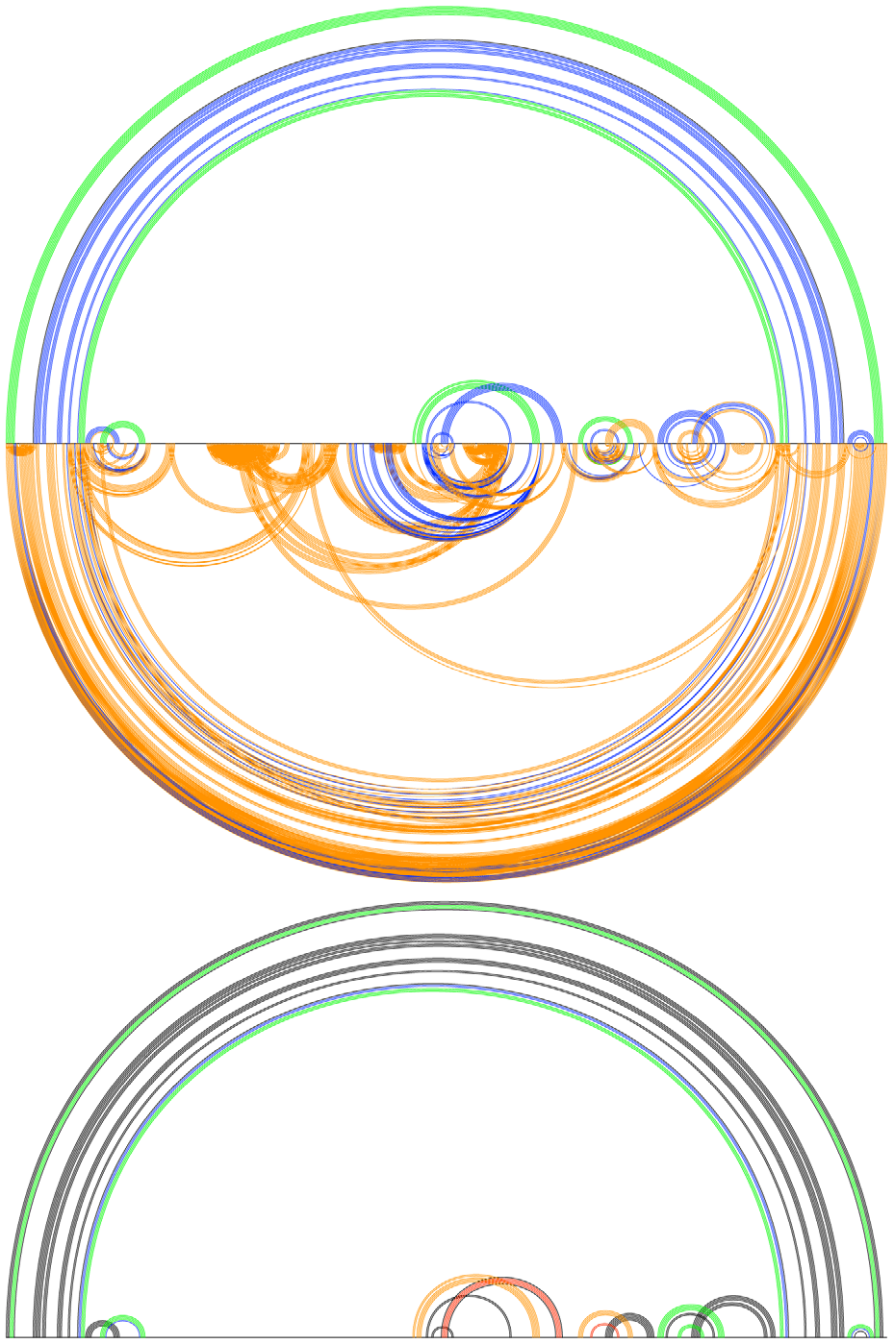
Comparison of Transat (top figure) and RNAalifold P (bottom figure) for the bacterial tmRNA, RF00023. The helices of the pseudo-knotted known structure for the bacterial tmRNA are correctly predicted by Transat. Transat also predicts several additional helices, but the region of the tmRNA sequences that contains the reading frame which ends in a translation stop signal is devoid of statistically significant transient helices supporting the hypothesis that the sequence in this region in the 5′ half of the RNA has been chosen to remain single-stranded and readily accessible. RNAalifold P predicts only a few of the helices of the known pseudo-knotted structure and no additional structural features, see also Figure 15. Please refer to the caption of Figure 16 for more information on arc-plots.

As opposed to Transat, the structural features of the thermodynamic ensemble predicted by RNAalifold P are not able to capture the cases where more than a single functional RNA secondary structures exist, see Figure 16 and Figure 17. If RNAalifold P predicts new base-pairs that are not part of the known reference structure(s), these tend to extend a known helices by one or two base-pairs to either side of the helix rather than correspond to entirely new helices as Transat does, see Figure 16, Figure 17, Figure 18 and Figures 6, 7 and 8 in Text S1. There are several cases where the thermodynamic ensemble predicted by RNAalifold P misses entire helices of the known RNA secondary structure(s), see Figure 16, Figure 17, Figure 18, Figure 19, Figure 20 and Figures 3, 4 and 9 in Text S1, and does not capture the pseudo-knotted structures well, see Figure 18, Figure 20 and Figure 3 in Text S1. In the few cases where RNAalifold P predicts novel helices, see Figure 19 and Figure 6 in Text S1, they are similar to a new helix predicted by Transat. Their ranking in terms of base-pairing probability, however, is often not in line with the p-value ranking of Transat and there exist cases where they differ from what Transat predicts, see Figures 7 and 10 in Text S1.

Overall, we thus conclude that the presence of multiple functional RNA secondary structures as well as of pseudo-knotted structures is better modelled using Transat than assuming a structural ensemble in thermodynamic equilibrium as predicted by RNAalifold P.

## Discussion

We devised Transat as a method to detect the statistically significant, conserved helices of functional RNA structures, including the helices of transient, pseudo-knotted and alternative structures as they are known to exist *in vivo*. As we explain in detail in the introduction, it is currently not possible to model the kinetic folding of RNA structures *in vivo* as function of the time as we not only lack many crucial details on the cellular environment that may influence the folding pathways (i.e. which molecules bind the RNA sequence in question when and where), but also because we currently have no adequate theoretical framework that would allow us to efficiently simulate the complex cellular environment using computational methods. We circumvent these conceptual problems by devising Transat as a comparative prediction method which takes a fixed multiple-sequence alignment of homologous RNA sequences and a tree quantifying their evolutionary relationship as input and detects evolutionarily conserved helices and estimates their statistical significance. By employing this comparative approach, we lose the ability to predict structural features of the cellular folding pathway(s) as function of the time and to detect species-specific structural features which may also be functionally important, but gain the ability to highlight statistically significant, functional helices that have been conserved *without actually having to model the cellular environment nor its evolution over time*.

Our comprehensive performance evaluation of Transat for a large and diverse data set (comprising 1126 multiple sequence alignments ranging from 100 to 1247 bp and comprising between 6 and 712 sequences) shows that Transat not only reliably detects the helices of known unique RNA reference structures, but that it also able to capture known pseudo-knotted structures as well as known alternative structural configurations. In addition to these known structural features, Transat predicts a number of distinct, novel helices of statistical significance. These may, for example, correspond to well-conserved structural features of a co-transcriptional folding pathway *in vivo* supporting the notion that homologous RNA sequence not only assume similar functional RNA structures, but also fold in a similar way. For some examples, the additional helices suggest a pseudo-knotted functional configuration, where only a pseudo-knot free RNA structure has been annotated so far. As we show for two examples, the predictions by Transat can also help identifying regions of an RNA sequence that are bound by other molecules and thus single-stranded because these are regions which are devoid of statistically significant helices. Detailed investigations show that Transat's predictions are robust with respect to alignment errors and modifications of the input tree and that its performance is fairly independent of the alignment length. Transat's performance is more correlated with the length of the input tree which is not surprising given that a certain degree of evolutionary diversity is required to observe pairs of co-varying alignment columns, where the base-pairing potential, but not necessarily the nucleotides forming the base-pairs has been conserved. We also find that the dominant structural features predicted by Transat typically do not coincide with those of the Boltzmann distribution of (pseudo-knot free) RNA secondary structures if we assume thermodynamic equilibrium. In particular, we find that the presence of known pseudo-knotted reference structures and of known alternative, functional RNA structures cannot be inferred from the Boltzmann distribution, i.e. by assuming thermodynamic equilibrium. This discrepancy may be partly attributed to the fact that the Boltzmann distribution does not include pseudo-knotted RNA structures, but overall confirms our expectation that there is *a priori* no good reason to assume that RNA sequences *in vivo* are in thermodynamic equilibrium or unbound by other molecules.

The Transat software is available from people.cs.ubc.ca/∼irmtraud/transat/. This web-page also contains information on the input and output files of this analysis as well as detailed documentation on how to use Transat. Users of Transat can rank the predicted helices according to their p-values with lower values implying higher statistical significance. Lab scientists seeking to confirm specific helices in dedicated experiments can prioritize their experiments by starting with the statistically most significant helices.

We hope that the predictions by Transat will enable more comprehensive and systematic studies of RNA folding pathways and alternative structural configurations and that they will provide useful input to the design and interpretation of future experiments. Whether the near future will bring more experimental insight into how RNA sequences fold *in vivo* depends to a large extent on the development of new experimental techniques that would allow us to observe an RNA sequence in its cellular environment.

Transat currently focuses on highly conserved structural features that are statistically significant, but ignores those that are functional, but only present in a small fraction of the input sequences. One possibility for future work is thus to extend Transat in order to also capture structural features that are only present in a few of all input sequences. As Transat already explicitly models the evolutionary relationship between all input sequences and the evolution of unpaired and base-paired nucleotides, this should be relatively straightforward to do. Another, more challenging possibility for future work is to take Transat beyond the required fixed input alignment. This is partly what the program SimulFold [32] addresses, but would need to done for individual helices and complemented by a corresponding procedure for estimating p-values.

## Supporting Information

Text S1 Supplementary Information and Figures
(0.96 MB PDF)
