# Supplementary material for: Transat—A Method for Detecting the Conserved Helices of Functional RNA Structures, Including Transient, Pseudo-Knotted and Alternative Structures
Source: PLoS Comput Biol. 2010 Jun 24;6(6):e1000823. doi: 10.1371/journal.pcbi.1000823 (PMC2891591; doi:10.1371/journal.pcbi.1000823)
Supplement: Text S1 — Supplementary Information and Figures (0.96 MB PDF) [file pcbi.1000823.s001.pdf]

Supplementary information for TRANSAT — a method for detecting the conserved helices of functional RNA structures, including transient, pseudo-knotted and alternative structures

Nicholas J. P. Wiebe and Irmtraud M. Meyer*, Centre for High-Throughput Biology & Department of Computer Science and Department of Medical Genetics, University of British Columbia, 2366 Main Mall, Vancouver, BC Canada V6T 1Z4, irmtraud.meyer@cantab.net

March 19, 2010

*corresponding author

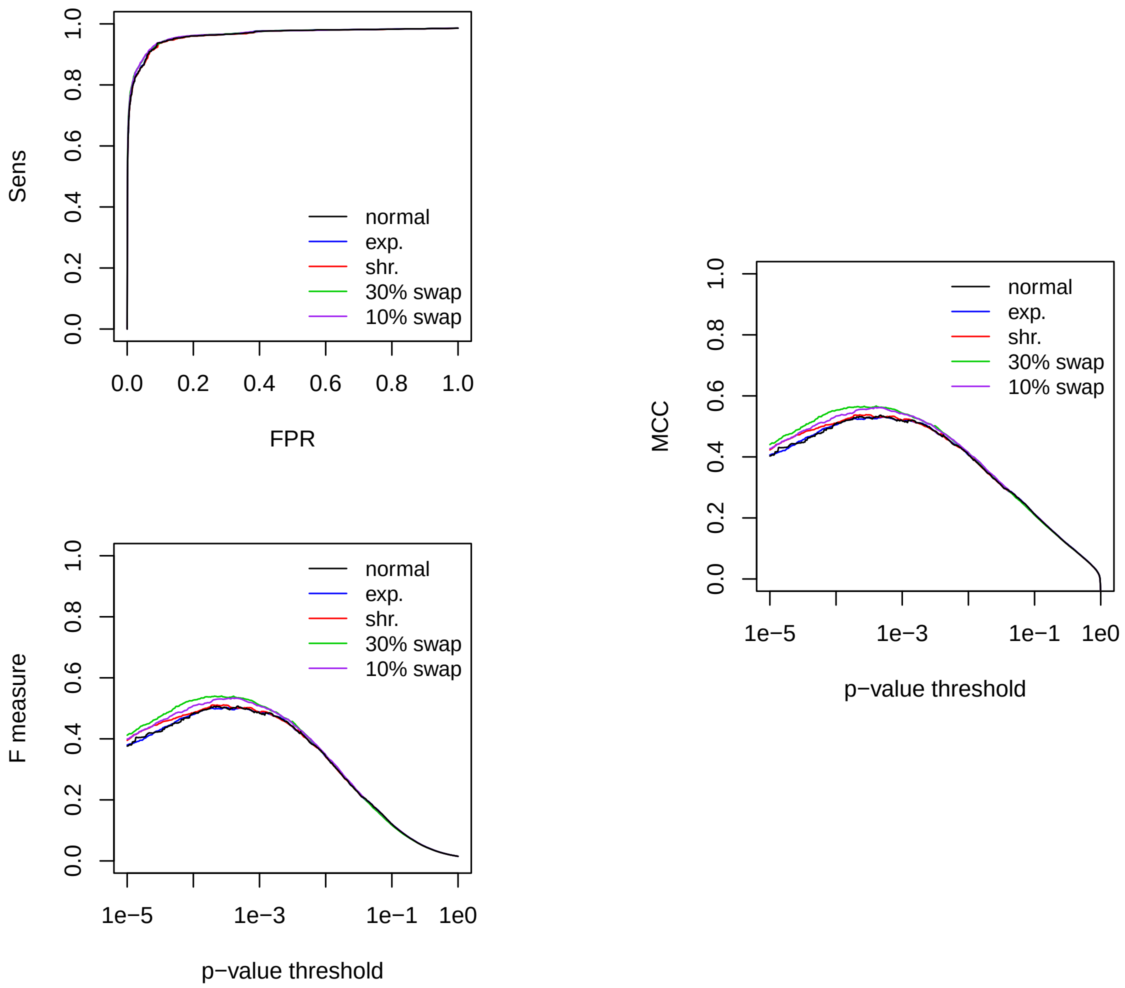

Figure 1: **Robustness of TRANSAT w.r.t. the quality of the input tree for the RFAM data set.** In order to investigate if minor changes of the input tree result in major changes of TRANSAT's performance, we modified the maximum-likelihood trees that we choose as input trees (see label "normal" above) in four ways: (1) we shrink all branch lengths in the tree by 10% (see label "shr."), (2) we expand them by 10% (see label "exp."), (3) we modify the topology of the tree by swapping 10% of its aunt-niece pairs (see label "10% swap") or (4) we swap 30% of all its aunt-niece pairs (see label "30% swap"). For modifications (3) and (4), we randomized each default tree five times and calculate the average performance of the resulting five trees. As the results above show, neither of the four types of tree modifications results in a significant change of TRANSAT's performance. TRANSAT is thus robust w.r.t. minor and major tree modifications.
Please refer to the main manuscript for the detailed definitions of all performance measures, see section "Performance evaluation". It is also interesting to note that the performance does not necessarily always decrease when the maximum-likelihood input trees (that we happen to choose as default input trees) are modified and that the overall effect of the change on a large data set can be positive. When investigating this effect in more detail, we found that most tree modifications tend to have a minor negative effect on the resulting performance for *individual* alignments in our RFAM set, but that it can occasionally result in a significantly improved performance. The maximum-likelihood trees that we use as default input trees to TRANSAT are thus a reasonable choice, but it may be possible to further improve TRANSAT's performance by choosing a different set of input trees.

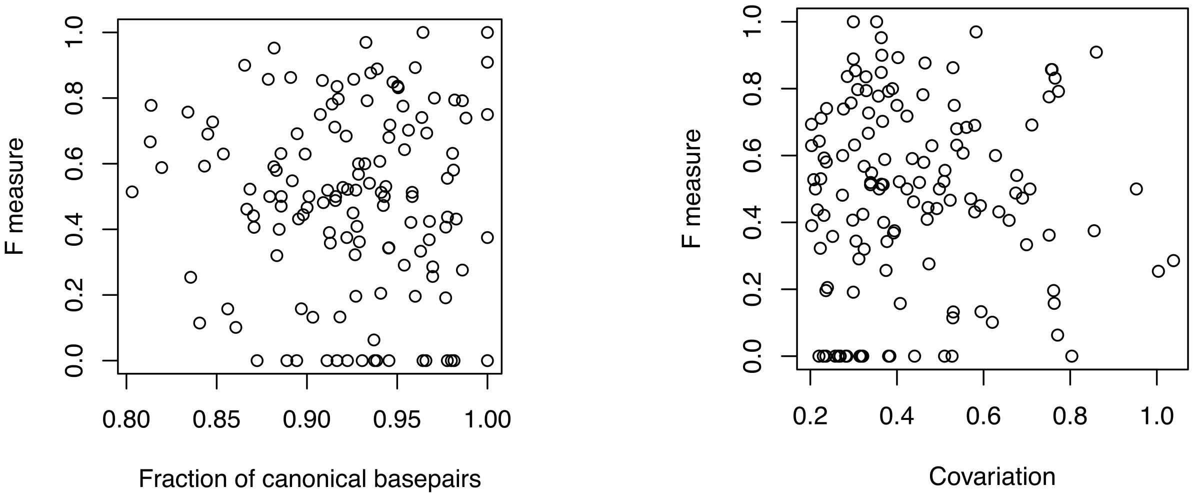

Figure 2: **Robustness of** Transat **w.r.t. the quality of the input alignment for the** Rfam **data set.** In order to investigate if Transat's performance strongly depends on the quality of the input alignment, we plot the F-measure as function of two alignment quality measures for all alignments of our Rfam data set: (1) the mean fraction of canonical base pairs (left figure) and (2) the covariation (right figure). The F-measure is defined as the harmonic mean of the sensitivity and the positive predictive value. Please refer to the main manuscript for the detailed definition, see section "Performance evaluation". The mean fraction of canonical base-pairs corresponds to the proportion of consensus base-pairs in the base-paired alignment columns of the consensus structure. The closer this fraction is to 1, the better the consensus structure is supported by all sequences in the seed alignment. The covariation measures the fraction of base-paired alignment columns that are supported by mutations which maintain the base-pairing ability, but alter the nucleotides forming the base-pair. As the results above show, there is no strong correlation between these the quality of the input alignment and Transat's performance. Unlike other prediction programs which require a fixed input alignment, we expect Transat to be fairly robust w.r.t. mis-alignments. This is because Transat derives candidate helices by mapping the helices for individual, i.e. unaligned sequences onto the fixed input alignment rather than by identifying them only directly in the alignment itself.

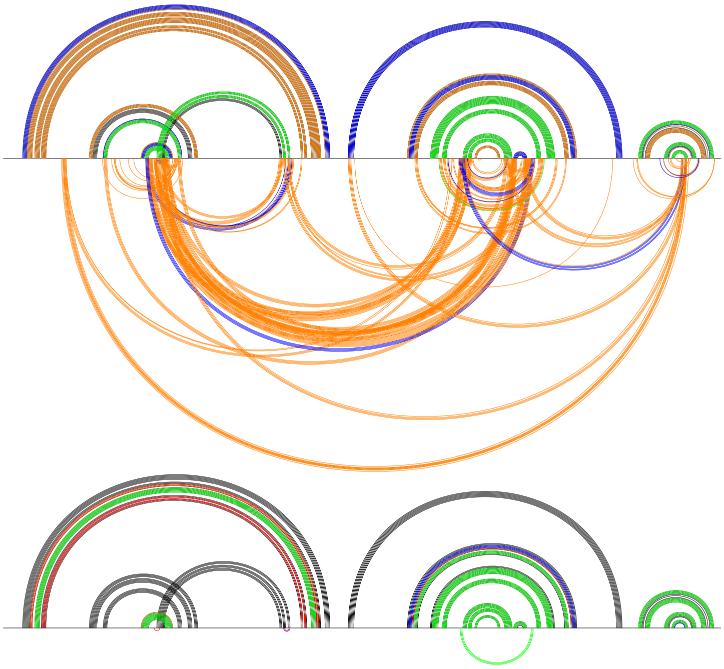

Figure 3: **Comparison of TRANSAT (top figure) and RNAALIFOLD P (bottom figure) for vertebrate telomerase RNA, RF00024.** TRANSAT most almost all helices of the known pseudo-knotted structure correctly as well as several new, statistically significant ones, whereas RNAALIFOLD P predicts only part of the known structure as well as one new helix which overlaps a new helix predicted by TRANSAT, see also Figure 12 in the manuscript. Please refer to the caption of Figure 16 in the manuscript for more information on arc-plots.

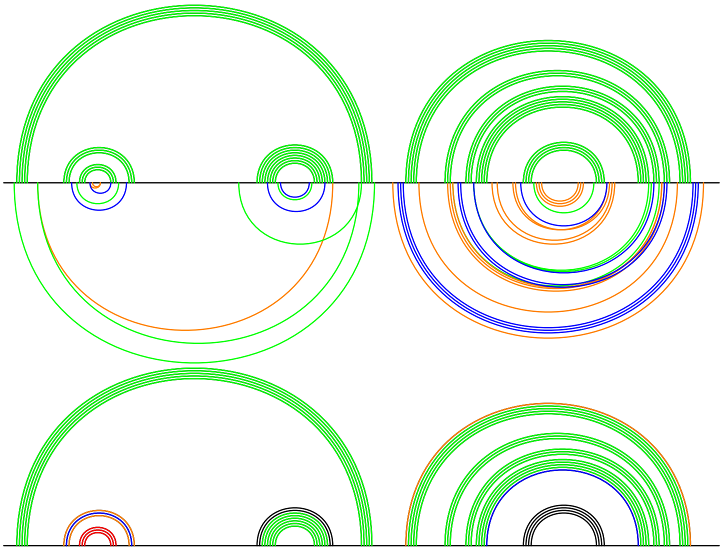

Figure 4: **Comparison of TRANSAT (top figure) and RNALIFOLD P (bottom figure) for ciliate telomerase RNA, RF00025.** The TRANSAT predictions indicate that the two hair-pins of the known ciliate structure form independently, whereas the formation of the hair-pins of the vertebrate structure shown in Figure 3 may involve long-range structural rearrangements. The predictions by RNALIFOLD P cover only part of the known structure and suggest no additional structural features. See also Figure 12 in the manuscript. Please refer to the caption of Figure 16 in the manuscript for more information on arc-plots.

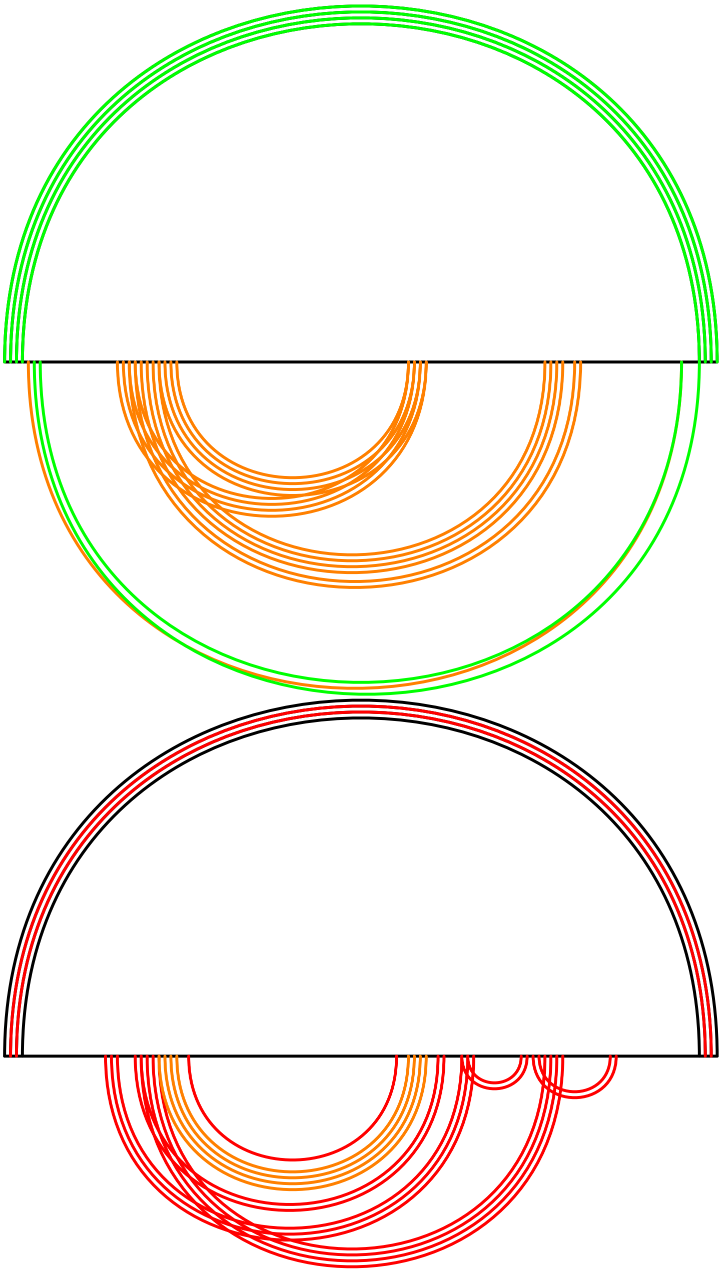

Figure 5: **Comparison of** TRANSAT **(top figure) and** RNALIFOLD P **(bottom figure) for the small nucleolar RNA snR76, RF01209.** Both TRANSAT and RNALIFOLD P predict several, mutually incompatible transient helices as well as the known, hairpin-like structure, but rank them differently accordingly to p-value or base-pairing probability. See also Figure 13 in the manuscript. Please refer to the caption of Figure 16 in the manuscript for more information on arc-plots.

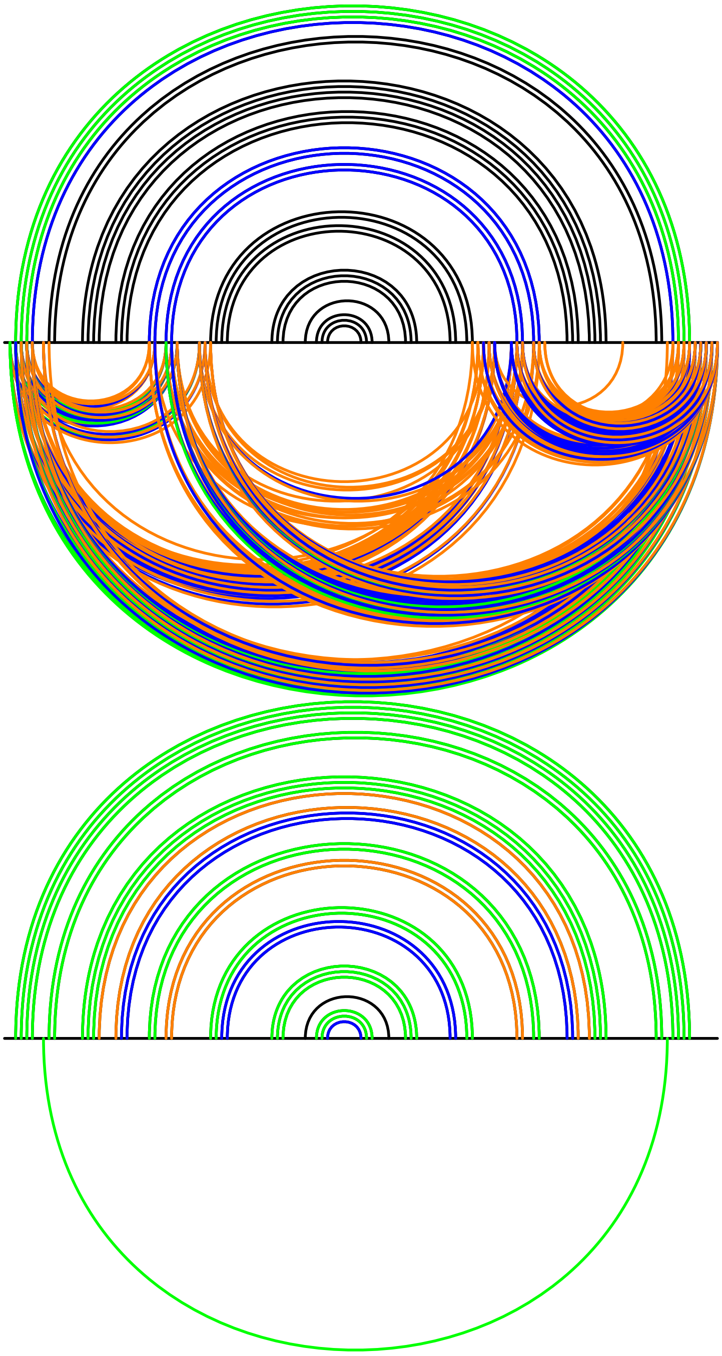

Figure 6: **Comparison of TRANSAT (top figure) and RNAALIFOLD P (bottom figure) for the bacterial signal recognition particle RNA, RF00169.**
The TRANSAT predictions indicate several, mutually incompatible transient helices and suggest a folding pathway involving several structural rearrangements, whereas RNAALIFOLD P predicts a larger fraction of the known base-pairs correctly and proposes only one new base-pair which is compatible with the known structure. See also Figure 13 in the manuscript. Please refer to the caption of Figure 16 in the manuscript for more information on arc-plots.

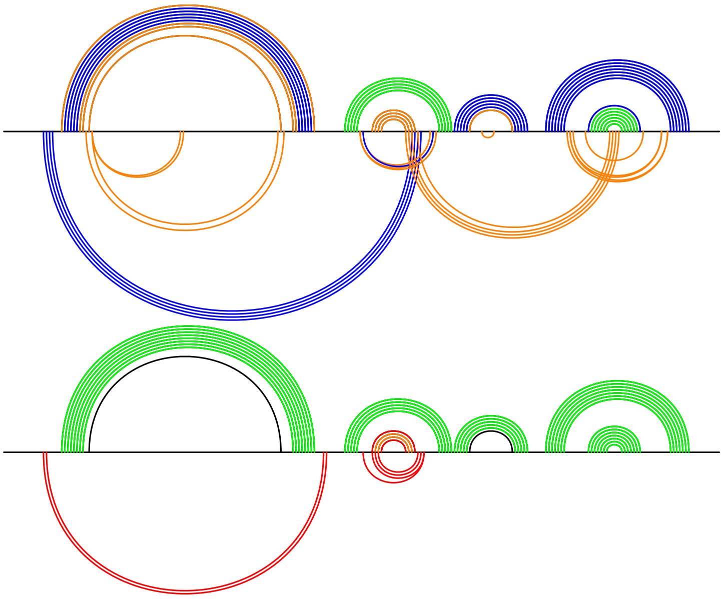

Figure 7: **Comparison of** Transat **(top figure) and** RNAlifold P **(bottom figure) for the glmS glucosamine-6-phosphate activated ribozyme, RF00234.** Transat predicts the helices of the known structures correctly and also provides strong statistical evidence (p-value $< 10^{-4}$) for additional helices that would render the known secondary structure pseudo-knotted, see the blue bottom-arcs. RNAlifold P also covers the known structure well and proposes fewer new helices which are different from the ones predicted by Transat and most of the new base-pairs are compatible with the known structure. See also Figure 14 in the manuscript. Please refer to the caption of Figure 16 in the manuscript for more information on arc-plots.

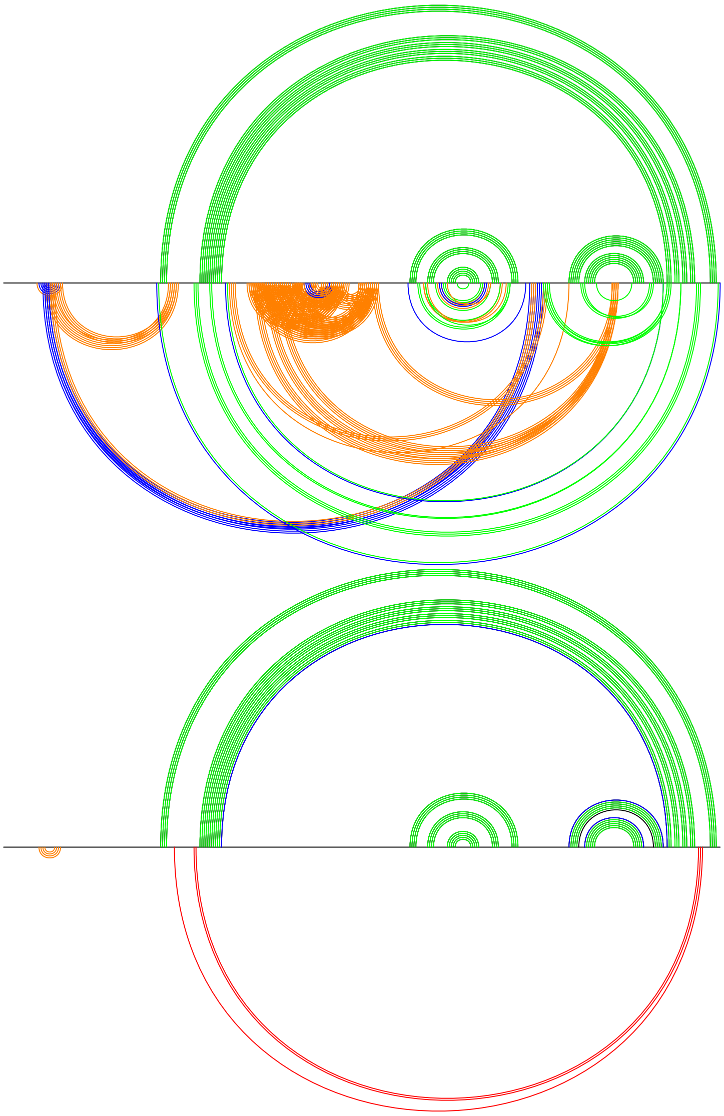

Figure 8: **Comparison of TRANSAT (top figure) and RNAALIFOLD P (bottom figure) for the small nucleolar RNA U3, RF00012.** TRANSAT predicts the helices of the known structure correctly and also provides strong statistical evidence (p-value $< 10^{-4}$) for several additional helices that would render the known secondary structure pseudo-knotted, see the blue bottom-arcs. RNAALIFOLD P also predicts the known structure well, but proposes fewer additional base-pair and less significance which are all compatible with the known structure. See also Figure 14 in the manuscript. Please refer to the caption of Figure 16 in the manuscript for more information on arc-plots.

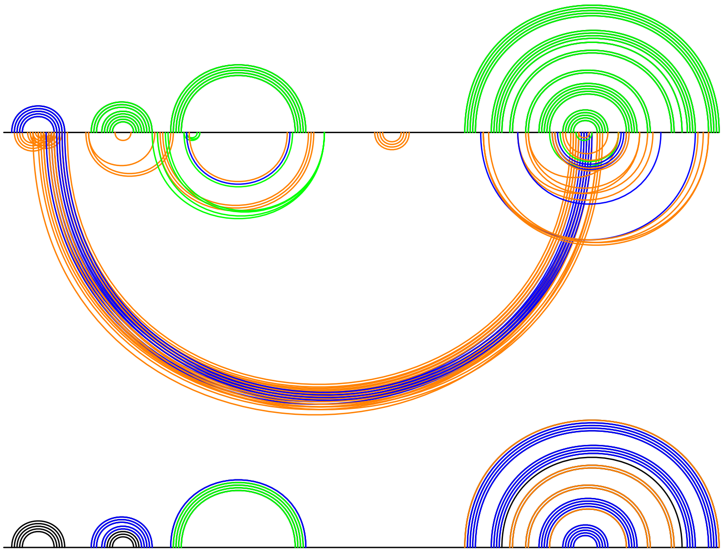

Figure 9: **Comparison of TRANSAT (top figure) and RNAALIFOLD P (bottom figure) for the U12 minor spliceosomal RNA, RF00007.**
TRANSAT predicts the helices of the known structures correctly and also provides strong statistical evidence (p-value $< 10^{-4}$) for an additional helix that would render the known secondary structure pseudoknotted, see the blue bottom-arcs. Note that this newly predicted helix is in competition with the most 5' helix that is part of the known RNA secondary structure. RNAALIFOLD P predicts only part of the known structure correctly and proposes no additional structure. See also Figure 14 in the manuscript. Please refer to the caption of Figure 16 in the manuscript for more information on arc-plots.

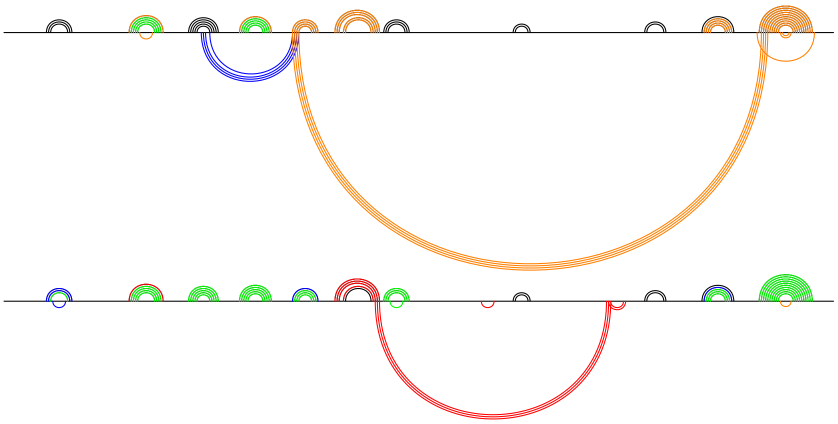

Figure 10: **Comparison of Transat (top figure) and RNAlifold P (bottom figure) for the CsrB/RsmB RNA family, RF00018.**
The CsrB/RsmB RNA is known to be bound by multiple copies of the CsrA protein. The RNA's known structure comprises only short range helices and Transat predicts only two transient structures for the entire 392 bp long alignment. Both findings support the hypothesis that protein binding occurs early during the folding of this RNA. The predictions by RNAlifold P cover the known structure even better and predict two additional helices which disagree with those predicted by Transat. See also Figure 15 in the manuscript. Please refer to the caption of Figure 16 in the manuscript for more information on arc-plots.
